# Synthesis and Antitumor Activity of Triazole-Containing Sorafenib Analogs

**DOI:** 10.3390/molecules22101759

**Published:** 2017-10-24

**Authors:** Wenjing Ye, Qi Yao, Simiao Yu, Ping Gong, Mingze Qin

**Affiliations:** Key Laboratory of Structure-Based Drug Design and Discovery (Shenyang Pharmaceutical University), Ministry of Education, 103 Wenhua Road, Shenyang 110016, China; yewenjing@syphu.edu.cn (W.Y.); 13664141520@163.com (Q.Y.); 18841498044m@sina.cn (S.Y.); gongpinggp@126.com (P.G.)

**Keywords:** binuclear Cu catalyst, sorafenib analogs, antitumor agents

## Abstract

Using a highly effective binuclear Cu complex as the catalyst, the 1,3-dipolar cycloaddition reactions between 16 alkynes and two azides were successfully performed and resulted in the production of 25 new triazole-containing sorafenib analogs. Several compounds were evaluated as potent antitumor agents. Among them, 4-(4-(4-(3-fluorophenyl)-1*H*-1,2,3-triazol-1-yl)phenoxy)-*N*-methylpicolinamide (**8f**) potently suppressed the proliferation of HT-29 cancer cells by inducing apoptosis and almost completely inhibited colony formation at a low micromolar concentration.

## 1. Introduction

Although significant progress has been achieved in the treatment of tumors, it is still a major health concern worldwide. Sorafenib is a well-known antitumor agent, which potently targets several kinases such as vascular endothelial growth factor receptor (VEGFR), platelet-derived growth factor receptor (PDGFR), stem cell factor receptor (c-Kit), and the downstream protein Raf [1]. It demonstrates excellent activity against a broad range of tumor types including renal, hepatocellular, and colorectal carcinomas [2,3,4]. However, the relative bioavailability of sorafenib is merely 38−49%, mainly due to its poor water solubility [5,6]. Thus, a feasible strategy for identifying antitumor agents could be the modification of sorafenib, paying equal attention to the potency and hydrophilicity of the derivatives.

The results of structure–activity relationship (SAR) analyses have indicated that the pyridine facilitates a suitable chemical interaction with Cys531 in a hinge region of B-Raf kinase, while the terminal phenyl ring occupies a hydrophobic pocket. The urea moiety connects both functional groups and forms important hydrogen-bond interactions with Glu500 and Asp593 [7,8,9,10]. Considerable studies were conducted on modification of the urea moiety, and potent compounds were identified by introducing an amide [11], rhodanine [12], or benzimidazole [13] group. From the viewpoint of medicinal chemistry, we considered that the urea moiety of sorafenib could be replaced by a 1,2,3-triazole fragment, which could act as a hydrogen-bond acceptor as well as a chemical linker between functional groups [14].

**Scheme 1:**
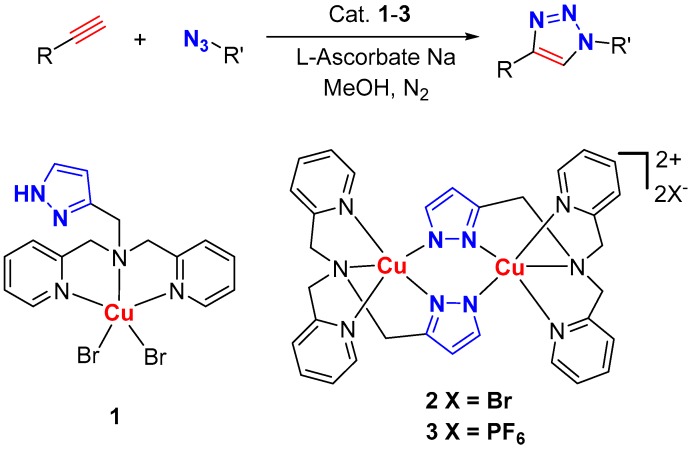
Cu complexes bearing an unsymmetrical bipyridine-pyrazole-amine ligand and application in CuAAC reaction.

The constructing of 1,2,3-triazole cycles has been intensively studied in recent years. The Cu(I)-catalyzed Huisgen 1,3-dipolar cycloaddition reaction between alkynes and azides (CuAAC) is known as one of the most efficient methods [15,16]. Numerous efficient catalytic systems have been developed for accelerating the catalytic process and decreasing the amount of metal catalyst needed over the past decade [17,18,19]. Very recently, we explored Cu complexes **1**–**3** bearing an unsymmetrical bipyridine-pyrazole-amine ligand and demonstrated their applications in the CuAAC reaction (Scheme 1) [20,21]. To the best of our knowledge, the binuclear complex **2** is currently one of the most active catalyst systems used in the CuAAC reaction.

**Figure 1:**
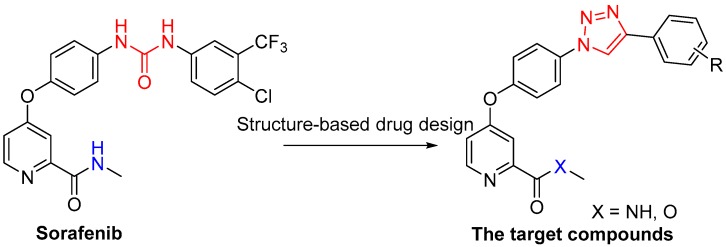
Design of novel sorafenib analogs.

Herein, we reported the application of our highly active catalyst (**2**) in the synthesis of novel sorafenib analogs containing a 1,2,3-triazole moiety (Figure 1). The 3-CF_3_-4-Cl phenyl moiety in the chemical structure of sorafenib was also modified and evaluated to identify an optimal substitution pattern. The antiproliferative activity of these compounds was evaluated in HT-29 and MCF-7 cancer cell lines. The biological evaluation led to the discovery of compound **8f**, which potently suppressed the proliferation of HT-29 cells by inducing apoptosis. Furthermore, **8f** effectively inhibited colony formation of HT-29 cells.

## 2. Results and Discussion

### 2.1. Chemistry

**Scheme 2:**
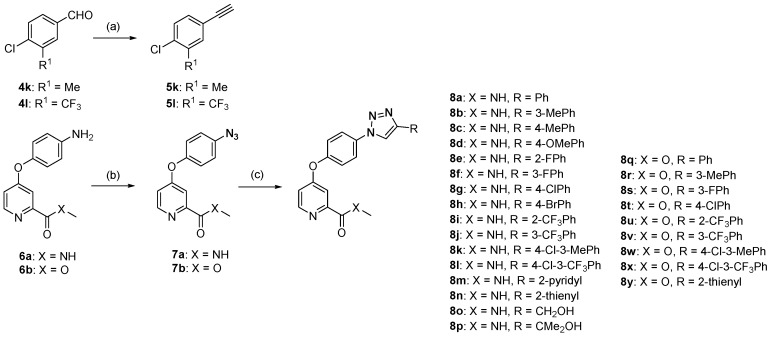
Synthesis route of the 1,2,3-triazole derivatives. Reagents and conditions: (**a**) K_2_CO_3_ 2.0 eq., Bestman–Ohira reagent 1.5 eq., MeOH, rt, 48 h, 74% yield in **5k**; 84% yield in **5l**; (**b**) HCl/NaNO_2_, 1.2 eq. NaN_3_, EtOAc, rt, 3 h, 61% yield in **7a**; 97% yield in **7b**; (**c**) appropriate alkyne, 0.1–0.3 mol % Cat. **2**, sodium l-ascorbate, MeOH, 25–50 °C, 16 h, 91–99% yields.

The preparation of compounds **8a**–**y** is described in Scheme 2. Most alkynes were obtained from commercial sources and were used without further purification. Alkynes **5k** and **5l** were prepared using the Seyferth–Gilbert reaction from the corresponding aldehydes **4k** and **4l**, which were treated with 1.5 equivalents of the Bestmann–Ohira reagent in the presence of 2.0 equivalents of potassium carbonate in methanol (MeOH) at room temperature [22]. The azides **7a** and **7b** were synthesized using a previously reported method [20]. Next, a library of triazolic derivatives was synthesized by cycloaddition reactions between various alkynes and azides using the binuclear complex **2** as the catalyst with a 0.1–0.3 mol % catalyst loading and sodium l-ascorbate as the reductant in MeOH (Scheme 2). Twenty-five triazoles were obtained with excellent isolated yields between 91 and 99%. The chemical structures of the triazoles were confirmed using nuclear magnetic resonance (NMR) and high-resolution mass spectrometry (HRMS). The object products were 1,4-disubtituted triazoles, which were determined by comparing the NMR spectra of **8a**–**y** with those of our previously reported triazoles [20].

### 2.2. Biological Evaluation

#### 2.2.1. In Vitro Antiproliferative Activity and SARs

The newly synthesized sorafenib analogs were screened for antiproliferative activity in two cancer cell lines, namely the HT-29 (human colorectal cancer) and MCF-7 (human breast cancer) using a standard 3-(4,5-dimethylthiazol-2-yl)-2,5-diphenyltetrazolium bromide (MTT) assay. Sorafenib was used as the positive control. The biological data, expressed as half-maximal inhibitory concentration (IC_50_) values, are listed in Table 1. Interestingly, we discovered that most compounds exhibited significant antiproliferative activity, especially in the HT-29 cell line. This selectivity in cell lines was quite consistent with that of sorafenib. Seven compounds, **8a**, **8e**, **8f**, **8i**, **8j**, **8m**, and **8n,** exhibited more potent activity than sorafenib did in inhibiting HT-29 cells. Notably, compounds **8e**, **8f**, and **8n** potently inhibited the proliferation of both selected cells, with IC_50_ values less than 1 μM, which highlighted their potential for further investigations. Compound **8f** was identified as the most promising compound, which exhibited a 26.5- and 80-fold increased potency compared with that of sorafenib in inhibiting the HT-29 and MCF-7 cell lines, respectively. In addition, compound **8f** possessed a ClogP of 3.6, which is quite suitable for drug development.

In addition, relatively clear SAR results were obtained for these compounds. An aromatic ring at the terminal might be indispensable for antiproliferative activity. For example, both compounds **8o** and **8p**, which have aliphatic substituents connecting the triazole moiety were radically inactive against the tested cancer cells. In contrast, most compounds (**8a**, **8b**, **8e**, **8f**, and **8i**–**8k**) bearing a phenyl group exhibited obvious activity. Compounds bearing a pyridine (**8m**) or thiophene (**8n**) fragment exhibited promising antiproliferative activity, indicating that the heterocyclic ring was also critical for this region.

Further investigations of the phenyl ring showed close SARs. Notably, a substituent at the para-position was detrimental to the activity of amide series of compounds. None of the related compounds showed antiproliferative activity against the HT-29 or MCF-7 cell line, even when an electron-withdrawing (**8g**, R = 4-ClPh and **8h**, R = 4-BrPh) or donating (**8c**, R = 4-MePh; **8d**, R = 4-OMePh) group was incorporated. For compound **8c**, shifting the methyl group to the meta-position produced compound **8b**, which exhibited significantly improved activity against the HT-29 cancer cells (IC_50_ = 8.53 μM). The results suggest that substituents at the meta-position were better tolerated. A similar observation was reported when the activity of **8e** (R = 2-FPh, IC_50_ = 0.84 μM) was compared with that of **8f** (R = 3-FPh, IC_50_ = 0.20 μM). However, the introduction of substituents at both the meta- and para-positions failed to generate potent compounds (**8k** and **8l**), which further confirmed that the para-position could not be substituted in this chemical series.

The analysis of the biological results showed that replacing the amide fragment at the 2-position of pyridine with an ester sharply decreased the activity. For example, compound **8a** (X = NH, R = Ph; IC_50_: 0.74 μM) exhibited potent inhibitory activity in HT-29 cells, but a 20.6-fold decrease in potency was reported for compound **8q** (X = O, R = Ph; IC_50_: 15.22 μM). Reduced potency was also observed when **8f** (X = NH, R = 3-FPh; IC_50_: 0.20 μM) was compared with **8s** (X = O, R = 3-FPh; IC_50_: 9.12 μM). These results indicate that a hydrogen-bond donor was necessary for activity, which was in line with the observation for sorafenib. In view of potent antiproliferative acitivity exhibited by **8f**, it was evaluated for cytotoxicity against normal cells derived from human embryonic kidney (HEK293T). Attractively, compound **8f** was less cytotoxic for HEK293T cells, with an IC_50_ value of 5.32 μM. These results indicated that compound **8f** was a good candidate for subsequent studies.

#### 2.2.2. Inhibition of Colony Formation by **8f**

**Figure 2:**
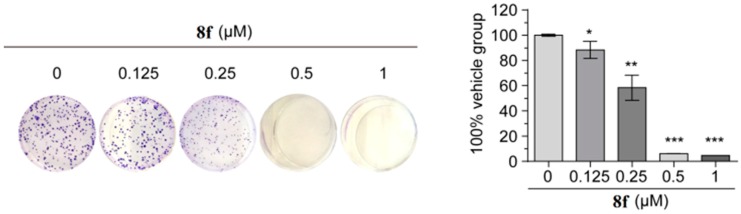
Representative images of colony formation of HT-29 cells treated with **8f**. Relative colony formation rate is shown as mean ± SEM. * *p* < 0.05, ** *p* < 0.01, and *** *p* < 0.001 versus vehicle group; *p*-values were calculated using an unpaired two-tailed Student’s *t*-test.

Considering the potent antiproliferative activity exhibited by **8f**, a colony formation assay was conducted for a more visual result. HT-29 cells were treated with **8f** at different concentrations (0, 0.125, 0.25, 0.5, and 1 μM) for 14 days. As shown in Figure 2, colony formation was significantly impaired in the **8f**-treated group at a concentration of 0.25 μM. Furthermore, treatment with 0.5 μM **8f** almost completely suppressed the colony formation.

#### 2.2.3. Induction of Cell Apoptosis by **8f**

**Figure 3:**
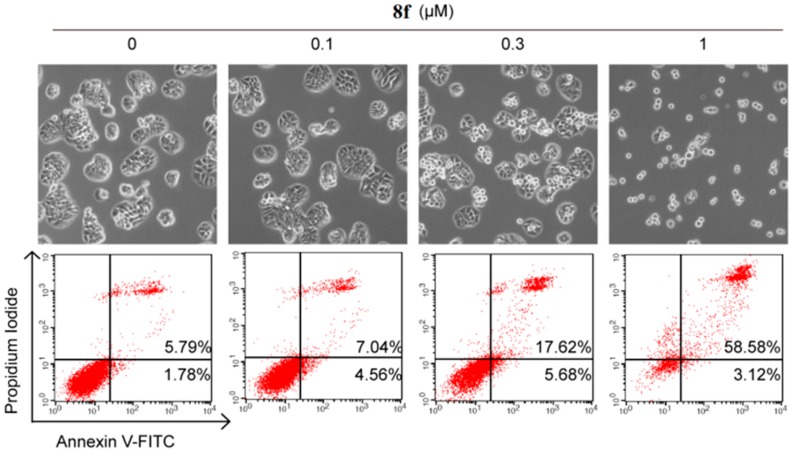
Effect of compound 8f on cell apoptosis in HT-29 cells.

The results of the MTT and colony formation assays clearly demonstrated that **8f** potently inhibited the proliferation of HT-29 cells. To determine whether **8f** acted by inducing apoptosis, a biparametric cytofluorimetric analysis using propidium iodide and annexin V-fluorescein isothiocyanate staining was performed. HT-29 cells were treated with **8f** at various concentrations (0, 0.1, 0.3, and 1 μM) for 72 h before staining and flow cytometry. As shown in Figure 3, the apoptotic rate of the HT-29 cells obviously increased from 7.57% (control) to 61.7% following treatment with 1 μM **8f**. The results suggest that **8f** induced significant apoptosis of HT-29 cells in a dose-dependent manner.

## 3. Experimental Section

### 3.1. General Information

The ^1^H- and ^13^C-NMR spectra were recorded on a Bruker AV-400 spectrometer (Bruker Co., Ltd., Zurich, Switzerland), with TMS as an internal standard. Melting points were determined on a Büchi Melting Point B-540 apparatus (Büchi Labortechnik, Flawil, Switzerland). The HRMS analysis was obtained on an Agilent Technologies 6530 Accurate-Mass Q-TOF LC/MS (Version Q-TOF B.05.01, Agilent Technologies Inc., Santa Clara, CA, USA). All the alkynes except **5k** and **5l** were purchased from commercial sources. Intermediates **5k** and **5l** were prepared according to a literature procedure. The characterization of compounds **8a**, **8h**, and **8q** have been reported in [20]. All of the solvents were purified according to the routine methods before being used.

### 3.2. General Procedure for the Synthesis of ***5k*** and ***5l***

Dimethyl-1-diazo-2-oxopropylphosphonate (3.6 mmol) was added to a solution of aldehyde **4** (3.0 mmol) and K_2_CO_3_ (6.0 mmol) in MeOH (3 mL) and stirring was continued for 24 h. The reaction mixture was diluted with Et_2_O (60 mL), washed with an aq solution (5%) of NaHCO_3_ (85 mL) and water (25 mL) successively and dried over Na_2_SO_4_. After filtration and evaporation of the solvent in vacuo, the resulting residue was purified by flash column chromatography to afford the corresponding alkyne (petroleum ether (PE) as an elution solvent).

*1-Chloro-4-ethynyl-2-methylbenzene* (**5k**). Yield: 74%, white solid. m.p.: 29.4–31.2 °C. ^1^H-NMR (600 MHz, DMSO-*d*_6_) δ 7.36 (d, 1H, *J* = 1.1 Hz, ArH), 7.29–7.24 (m, 2H, ArH), 3.07 (s, 1H, C≡CH), 2.25 (s, 3H, CH_3_).

*1-Chloro-4-ethynyl-2-(trifluoromethyl)benzene* (**5l**). Yield: 84%, white solid. m.p.: 29.6–30.1 °C. ^1^H-NMR (600 MHz, DMSO-*d*_6_) δ 7.80 (d, 1H, *J* = 1.8 Hz, ArH), 7.58 (dd, 1H, *J* = 8.3, 1.9 Hz, ArH), 7.48 (d, 1H, *J* = 8.3 Hz, ArH), 3.19 (s, 1H, C≡CH).

### 3.3. General Procedure for 2-Catalyzed 1,3-Dipolar Cycloaddition Reactions

Under N_2_ atmosphere, the mixture of alkyne (0.3 mmol), azide (0.3 mmol), Na ascorbate (0.006 mmol, 2 mol %), and 0.3 × 10^−3^ M solution of catalyst **2** in MeOH (0.1–0.3 mol %) was stirred in a 10 mL Schlenk tube at 25 °C for 16 h. Evaporation of the solvents followed by purification by short column chromatography on silica gel provided the desired 1,4-disubstituted triazole product. The unreacted alkyne and azide were first eluted out with 3/1 petroleum ether (PE)/ethyl acetate, and the pure 1,4-disubstituted triazole product was then obtained by elution with pure ethyl acetate.

*N-Methyl-4-(4-(4-(m-tolyl)-1H-1,2,3-triazol-1-yl)phenoxy)picolinamide* (**8b**). Yield: 99%, white solid. m.p.: 185–187 °C. ^1^H-NMR (600 MHz, DMSO-*d*_6_) δ 9.33 (s, 1H, triazole-H), 8.82 (q, 1H, *J* = 4.8 Hz, NH), 8.57 (d, 1H, *J* = 5.6 Hz, ArH), 8.10 (dt, 2H, *J* = 8.9, 2.3 Hz, ArH), 7.79 (s, 1H, ArH), 7.75 (d, 1H, *J* = 7.7 Hz, ArH), 7.53 (dt, 2H, *J* = 8.9, 2.2 Hz, ArH), 7.49 (d, 1H, *J* = 2.6 Hz, ArH), 7.40 (t, 1H, *J* = 7.7 Hz, ArH), 7.27 (dd, 1H, *J* = 5.6, 2.6 Hz, ArH), 7.22 (d, 1H, *J* = 7.5 Hz, ArH), 2.80 (d, 3H, *J* = 4.9 Hz, NCH_3_), 2.39 (s, 3H, CCH_3_). ^13^C-NMR (151 MHz, DMSO-*d*_6_) δ 165.18, 163.72, 153.28, 152.66, 150.67, 147.49, 138.21, 134.15, 130.13, 128.96, 125.91, 122.55, 122.37, 122.27, 119.80, 114.59, 109.34, 26.06, 21.12. HRMS Calcd for C_22_H_19_N_5_O_2_ [M]: 385.1539; found for C_22_H_19_N_5_O_2_ [M]: 385.1532.

*N-Methyl-4-(4-(4-(p-tolyl)-1H-1,2,3-triazol-1-yl)phenoxy)picolinamide* (**8c**). Yield: 99%, white solid. m.p.: 195–197 °C. ^1^H-NMR (600 MHz, DMSO-*d*_6_) δ 9.30 (s, 1H, triazole-H), 8.84 (q, 1H, *J* = 4.4 Hz, NH), 8.58 (d, 1H, *J* = 5.6 Hz, ArH), 8.10 (dt, 2H, *J* = 10.0, 3.2 Hz, ArH), 7.85 (d, 2H, *J* = 8.0 Hz, ArH), 7.54–7.49 (m, 3H, ArH), 7.33 (d, 2H, *J* = 7.9 Hz, ArH), 7.27 (dd, 1H, *J* = 5.6, 2.6 Hz, ArH), 2.80 (d, 3H, *J* = 4.9 Hz, NHCH_3_), 2.36 (s, 3H, CCH_3_). ^13^C-NMR (151 MHz, DMSO-*d*_6_) δ 165.18, 163.70, 153.25, 152.65, 150.66, 147.45, 137.69, 134.17, 129.59, 127.44, 125.30, 122.36, 122.30, 119.46, 114.58, 109.32, 26.05, 20.90. HRMS Calcd for C_22_H_19_N_5_O_2_ [M]: 385.1539; found for C_22_H_19_N_5_O_2_ [M]: 385.1558.

*4-(4-(4-(4-Methoxyphenyl)-1H-1,2,3-triazol-1-yl)phenoxy)-N-methylpicolinamide* (**8d**). Yield: 97%, white solid. m.p.: 215–217 °C. ^1^H-NMR (600 MHz, DMSO-*d*_6_) δ 9.24 (s, 1H, triazole-H), 8.83 (d, 1H, *J* = 4.8 Hz, NH), 8.58 (d, 1H, *J* = 5.6 Hz, ArH), 8.10 (dt, 2H, *J* = 8.9, 3.2 Hz, ArH), 7.90 (dt, 2H, *J* = 8.8, 2.8 Hz, ArH), 7.54–7.49 (m, 3H, ArH), 7.27 (dd, 1H, *J* = 8.0, 2.6 Hz, ArH), 7.10 (dt, 2H, *J* = 8.8, 2.8 Hz, ArH), 3.82 (s, 3H, OCH_3_), 2.81(d, 3H, *J* = 4.9 Hz, NCH_3_). ^13^C-NMR (151 MHz, DMSO-*d*_6_) δ 165.20, 163.72, 159.35, 153.21, 152.65, 150.68, 147.36, 134.21, 126.75, 122.75, 122.37, 122.26, 118.89, 114.58, 114.48, 109.33, 55.24, 26.07. HRMS Calcd for C_22_H_19_N_5_O_3_ [M]: 401.1488; found for C_22_H_19_N_5_O_3_ [M]: 401.1471.

*4-(4-(4-(2-Fluorophenyl)-1H-1,2,3-triazol-1-yl)phenoxy)-N-methylpicolinamide* (**8e**). Yield: 98%, white solid. m.p.: 182–183 °C. IR (KBr, cm^−1^): 3395.0, 1684.6, 1631.3, 1604.1, 1566.3, 1533.9, 1512.4, 901.8, 859.3, 837.4. ^1^H-NMR (600 MHz, DMSO-*d*_6_) δ 9.14 (d, 1H, *J* = 2.9 Hz, triazole-H), 8.83 (q, 1H, *J* = 4.4 Hz, NH), 8.57(d, 1H, *J* = 5.6 Hz, ArH), 8.21–8.14 (m, 3H, ArH), 7.52–7.46 (m, 4H, ArH), 7.42–7.37 (m, 2H, ArH), 7.27 (dd, 1H, *J* = 5.5, 2.6 Hz, ArH), 2.80 (d, 3H, *J* = 4.9 Hz, CH_3_). ^13^C-NMR (151 MHz, DMSO-*d*_6_) δ 165.17, 163.72, 159.43, 157.79, 153.43, 152.66, 150.68, 141.01, 140.99, 134.01, 130.23, 130.18, 127.76, 127.74, 125.09, 125.07, 122.78, 122.29, 122.09, 122.02, 118.00, 117.91, 116.27, 116.13, 114.61, 109.34, 26.06. HRMS Calcd for C_21_H_17_N_5_O_2_ [M]: 389.1288; found for C_21_H_17_N_5_O_2_ [M]: 389.1304.

*4-(4-(4-(3-Fluorophenyl)-1H-1,2,3-triazol-1-yl)phenoxy)-N-methylpicolinamide* (**8f**). Yield: 99%, white solid. m.p.: 209–210 °C. IR (KBr, cm^−1^): 3403.3, 1676.7, 1630.7, 1610.4, 1589.8, 1568.9, 1538.8, 1514.7, 897.2, 862.4, 836.4. ^1^H-NMR (600 MHz, DMSO-*d*_6_) δ 9.42 (s, 1H, triazole-H), 8.83 (q, 1H, NH), 8.58 (d, 1H, *J* = 5.6 Hz, ArH), 8.09 (d, 2H, *J* = 8.9 Hz, ArH), 7.82 (d, 1H, *J* = 7.8 Hz, ArH), 7.76 (d, 1H, *J* = 10.1 Hz, ArH), 7.59–7.52 (m, 3H, ArH), 7.49 (d, 1H, *J* = 2.6 Hz, ArH), 7.27–7.22 (m, 2H, ArH), 2.80 (d, 3H, *J* = 4.9 Hz, CH_3_). ^13^C-NMR (150 MHz, DMSO-*d*_6_) δ 165.15, 163.72, 163.45, 161.83, 153.44, 152.67, 150.69, 146.29 (d, *J* = 2.7 Hz), 134.03, 132.61 (d, *J* = 8.6 Hz), 132.55 (d, *J* = 8.5 Hz), 131.30, 131.25, 122.44 (d, *J* = 2.8 Hz), 121.41 (d, *J* = 2.5 Hz), 120.71 (d, *J* = 2.6 Hz), 115.13, 114.99, 114.63, 112.03, 111.88, 109.36, 26.07. HRMS Calcd for C_21_H_16_FN_5_O_2_ [M]^+^: 389.1288; found for C_21_H_16_FN_5_O_2_ [M + H]^+^: 390.1355.

*4-(4-(4-(4-Chlorophenyl)-1H-1,2,3-triazol-1-yl)phenoxy)-N-methylpicolinamide* (**8g**). Yield: 94%, white solid. m.p.: 244–246 °C. ^1^H-NMR (600 MHz, DMSO-*d*_6_) δ 9.40 (s, 1H, triazole-H), 8.83 (d, 1H, *J* = 4.8 Hz, NH), 8.58 (d, 1H, *J* = 5.6 Hz, ArH), 8.09 (d, 2H, *J* = 8.9 Hz, ArH), 7.98 (d, 2H, *J* = 8.5 Hz, ArH), 7.60 (d, 2H, *J* = 8.5 Hz, ArH), 7.54 (d, 2H, *J* = 8.9 Hz, ArH), 7.49 (d, 1H, *J* = 2.5 Hz, ArH), 7.28 (dd, 1H, *J* = 5.6, 2.6 Hz, ArH), 2.81 (d, 3H, *J* = 4.9 Hz, CH_3_). ^13^C-NMR (151 MHz, DMSO-*d*_6_) δ 165.16, 163.72, 153.39, 152.66, 150.69, 146.30, 134.06, 132.77, 129.17, 129.14, 127.05, 122.42, 122.40, 120.33, 120.31, 114.62, 109.35, 26.07. HRMS Calcd for C_21_H_16_ClN_5_O_2_ [M]: 405.0993; found for C_21_H_16_ClN_5_O_2_ [M]: 405.0991.

*N-Methyl-4-(4-(4-(2-(trifluoromethyl)phenyl)-1H-1,2,3-triazol-1-yl)phenoxy)picolinamide* (**8i**). Yield: 99%, white solid. m.p.: 51–54 °C. ^1^H-NMR (600 MHz, DMSO-*d*_6_) δ 9.07 (s, 1H, triazole-H), 8.83 (q, 1H, *J* = 4.8 Hz, NH), 8.58 (d, 1H, *J* = 5.6 Hz, ArH), 8.14 (dt, 2H, *J* = 8.9, 3.2 Hz, ArH), 7.94 (d, 1H, *J* = 8.0 Hz, ArH), 7.84–7.83 (m, 2H, ArH), 7.73–7.71 (m, 1H, ArH), 7.53–7.50 (m, 3H, ArH), 7.27 (dd, 1H, *J* = 4.9, 2.5 Hz, ArH), 2.81 (d, 1H, *J* = 4.9 Hz, CH_3_). ^13^C-NMR (151 MHz, DMSO-*d*_6_) δ 165.17, 163.71, 153.45, 152.67, 150.68, 144.84, 133.90, 132.76, 132.22, 129.36, 128.98, 127.08 (q, *J* = 30.2 Hz), 126.51 (q, *J* = 5.5 Hz), 124.89, 123.08, 122.59, 122.41, 114.59, 109.37, 26.06. HRMS Calcd for C_21_H_16_F_3_N_5_O_2_ [M]: 439.1256; found for C_21_H_16_F_3_N_5_O_2_ [M]: 439.1245.

*N-Methyl-4-(4-(4-(3-(trifluoromethyl)phenyl)-1H-1,2,3-triazol-1-yl)phenoxy)picolinamide* (**8j**). Yield: 99%, white solid. m.p.: 216–218 °C. IR (KBr, cm^−1^): 3391.6, 1678.7, 1594.3, 1573.2, 1542.9, 835.8, 800.3. ^1^H-NMR (600 MHz, DMSO-*d*_6_) δ 9.56 (s, 1H, triazole-H), 8.83 (d, 1H, *J* = 4.9 Hz, NH), 8.59 (d, 1H, *J* = 5.6 Hz, ArH), 8.29 (d, 2H, *J* = 4.9 Hz, ArH), 8.11 (dt, 2H, *J* = 8.9, 2.0 Hz, ArH), 7.78–7.77 (m, 2H, ArH), 7.56 (dt, 2H, *J* = 8.9, 2.0 Hz, ArH), 7.50 (d, 1H, *J* = 2.5 Hz, ArH), 7.28 (dd, 1H, *J* = 5.6, 2.6 Hz, ArH), 2.81 (d, 3H, *J* = 4.9 Hz, CH_3_). ^13^C-NMR (151 MHz, DMSO-*d*_6_) δ 165.14, 163.71, 153.47, 152.67, 150.69, 145.96, 134.00, 131.32, 130.36, 130.23 (q, *J_F_* = 31.8 Hz), 129.11, 126.87, 125.06, 124.84, 124.81, 123.26, 122.42, 122.38, 121.71, 121.68, 121.45, 120.96, 120.94, 114.64, 109.38, 26.06. HRMS Calcd for C_21_H_16_F_3_N_5_O_2_ [M]: 439.1256; found for C_21_H_16_F_3_N_5_O_2_ [M]: 439.1245.

*4-(4-(4-(4-Chloro-3-methylphenyl)-1H-1,2,3-triazol-1-yl)phenoxy)-N-methylpicolinamide* (**8k**). Yield: 99%, white solid. m.p.: 237–238 °C. ^1^H-NMR (600 MHz, DMSO-*d*_6_) δ 9.38 (s, 1H, triazole-H), 8.83 (d, 1H, *J* = 4.3 Hz, NH), 8.58 (d, 1H, *J* = 5.4 Hz, ArH), 8.09 (d, 1H, *J* = 8.5 Hz, ArH), 7.96 (s, 1H, ArH), 7.80 (d, 1H, *J* = 7.8 Hz, ArH), 7.57–7.49 (m, 4H, ArH), 7.28 (d, 1H, *J* = 2.9 Hz, ArH), 2.81 (d, 3H, *J* = 4.6 Hz, NCH_3_), 2.43 (s, 3H, CCH_3_). ^13^C-NMR (151 MHz, DMSO-*d*_6_) δ 165.16, 163.71, 153.37, 152.66, 150.69, 146.42, 136.19, 134.07, 133.00, 129.59, 129.16, 127.93, 124.51, 122.39, 122.34, 120.20, 114.62, 109.35, 26.06, 19.75. HRMS Calcd for C_22_H_18_ClN_5_O_2_ [M]: 419.1149; found for C_22_H_18_ClN_5_O_2_ [M]: 419.1137.

*4-(4-(4-(4-Chloro-3-(trifluoromethyl)phenyl)-1H-1,2,3-triazol-1-yl)phenoxy)-N-methylpicolinamide* (**8l**). Yield: 98%, white solid. m.p.: 226–228 °C. ^1^H-NMR (600 MHz, DMSO-*d*_6_) δ 9.58 (s, 1H, triazole-H), 8.83 (d, 1H, *J* = 4.9 Hz, NH), 8.59 (d, 1H, *J* = 5.6 Hz, ArH), 8.38 (d, 1H, *J* = 1.9 Hz, ArH), 8.27 (dd, 1H, *J* = 8.3, 1.8 Hz, ArH), 8.10 (dt, 2H, *J* = 8.9, 3.2 Hz, ArH), 7.91 (d, 1H, *J* = 8.4 Hz, ArH), 7.56–7.50 (m, 3H, ArH), 7.28 (dd, 1H, *J* = 5.6, 2.6 Hz, ArH), 2.81 (d, 3H, *J* = 4.9 Hz, CH_3_). ^13^C-NMR (151 MHz, DMSO-*d*_6_) δ 165.12, 163.71, 153.54, 152.67, 150.69, 145.06, 133.91, 132.62, 130.45, 130.09, 129.93, 127.52, 127.31, 124.27, 124.23, 123.70, 122.42, 122.41, 121.89, 121.28, 121.26, 114.65, 109.38, 26.06. HRMS Calcd for C_22_H_15_ClF_3_N_5_O_2_ [M]: 473.0866; found for C_22_H_15_ClF_3_N_5_O_2_ [M]: 473.0883.

*N-Methyl-4-(4-(4-(pyridin-2-yl)-1H-1,2,3-triazol-1-yl)phenoxy)picolinamide* (**8m**). Yield: 94%, white solid. m.p.: 200–201 °C. ^1^H-NMR (600 MHz, DMSO-*d*_6_) δ 9.40 (s, 1H, triazole-H), 8.84 (q, 1H, *J* = 4.5 Hz, NH), 8.68 (d, 1H, *J* = 4.7 Hz, ArH), 8.58 (d, 1H, *J* = 5.6 Hz, ArH), 8.19–8.14 (m, 3H, ArH), 7.98 (dt, 1H, *J* = 7.7, 1.6 Hz, ArH), 7.52 (m, 3H, ArH), 7.43 (dd, 1H, *J* = 7.4, 4.9 Hz, ArH), 7.28 (dd, 1H, *J* = 5.5, 2.6 Hz, ArH), 2.81, 2.80 (d, 3H, *J* = 4.9 Hz, CH_3_). ^13^C-NMR (151 MHz, DMSO-*d*_6_) δ 165.16, 163.72, 153.41, 152.65, 150.67, 149.73, 149.48, 148.29, 137.41, 134.05, 123.43, 122.54, 122.29, 121.52, 121.51, 119.86, 114.63, 109.36, 26.07. HRMS Calcd for C_20_H_16_N_6_O_2_ [M]: 372.1335; found for C_20_H_16_N_6_O_2_ [M]: 372.1340.

*N-methyl-4-(4-(4-(thiophen-2-yl)-1H-1,2,3-triazol-1-yl)phenoxy)picolinamide* (**8n**). Yield: 92%, white solid. m.p.: 185–188 °C. IR (KBr, cm^−1^): 3381.7, 1677.0, 1631.4, 1587.3, 1571.9, 1530.8, 1511.8, 849.6, 838.6, 816.1. ^1^H-NMR (600 MHz, DMSO-*d*_6_) δ 9.26 (s, 1H, triazole-H), 8.83 (d, 1H, *J* = 4.8 Hz, NH), 8.58 (d, 1H, *J* = 5.6 Hz, ArH), 8.09 (d, 2H, *J* = 8.9 Hz, ArH), 7.63 (dd, 1H, *J* = 5.0, 0.8 Hz, ArH), 7.54–7.49 (m, 4H, ArH), 7.27 (dd, 1H, *J* = 5.5, 2.6 Hz, ArH), 7.21 (dd, 1H, *J* = 4.9, 3.6 Hz, ArH), 2.81 (d, 3H, *J* = 4.9 Hz, CH_3_). ^13^C-NMR (151 MHz, DMSO-*d*_6_) δ 165.14, 163.69, 153.39, 152.65, 150.66, 142.79, 133.95, 132.30, 128.06, 126.01, 124.68, 122.43, 122.34, 119.14, 114.60, 109.35, 26.05. HRMS Calcd for C_19_H_15_N_5_O_2_S [M]: 377.0946; found for C_19_H_15_N_5_O_2_S [M]: 377.0938.

*4-(4-(4-(Hydroxymethyl)-1H-1,2,3-triazol-1-yl)phenoxy)-N-methylpicolinamide* (**8o**). Yield: 92%, white solid. m.p.: 191–192 °C. ^1^H-NMR (600 MHz, DMSO-*d*_6_) δ 8.82 (d, 1H, *J* = 4.9 Hz, NH), 8.74 (s, 1H, triazole-H), 8.57 (d, 2H, *J* = 5.6 Hz, ArH), 8.06 (dt, 2H, *J* = 8.9, 3.2 Hz, ArH), 7.49–7.46 (m, 3H, ArH), 7.26 (dd, 1H, *J* = 5.6, 2.6 Hz, ArH), 5.39 (t, 1H, *J* = 5.6 Hz, OH), 4.64 (d, 2H, *J* = 5.5 Hz, CH_2_), 2.80 (d, 3H, *J* = 4.9 Hz, CH_3_). ^13^C-NMR (151 MHz, DMSO-*d*_6_) δ 165.18, 163.72, 153.11, 152.64, 150.66, 149.25, 134.27, 122.29, 122.26, 121.23, 114.58, 109.32, 54.98, 26.06. HRMS Calcd for C_16_H_15_N_5_O_3_ [M]: 325.1175; found for C_16_H_15_N_5_O_3_ [M]: 325.1191.

*4-(4-(4-(2-Hydroxypropan-2-yl)-1H-1,2,3-triazol-1-yl)phenoxy)-N-methylpicolinamide* (**8p**). Yield: 99%, white solid. m.p.: 150–152 °C. ^1^H-NMR (600 MHz, DMSO-*d*_6_) δ 8.83 (q, 1H, *J* = 4.5 Hz, NH), 8.65 (s, 1H, triazole-H), 8.57 (d, 1H, *J* = 5.6 Hz, ArH), 8.06 (dt, 2H, *J* = 8.9, 3.2 Hz, ArH), 7.48–7.45 (m, 3H, ArH), 7.25 (dd, 1H, *J* = 5.6, 2.6 Hz, ArH), 5.28 (s, 1H, OH), 2.81 (d, 3H, *J* = 4.9 Hz, NCH_3_), 1.55 (s, 6H, C(CH_3_)_2_). ^13^C-NMR (151 MHz, DMSO-*d*_6_) δ 165.19, 163.72, 157.03, 153.01, 152.64, 150.66, 134.34, 122.25, 122.17, 119.10, 114.57, 109.33, 67.02, 30.60, 26.06. HRMS Calcd for C_18_H_19_N_5_O_3_ [M]: 353.1488; found for C_18_H_19_N_5_O_3_ [M]: 353.1504.

*Methyl 4-(4-(4-(m-tolyl)-1H-1,2,3-triazol-1-yl)phenoxy)picolinate* (**8r**). Yield: 98%, white solid. m.p.: 147–149 °C. ^1^H-NMR (600 MHz, DMSO-*d*_6_) δ 9.34 (s, 1H, triazole-H), 8.65 (d, 1H, *J* = 5.5 Hz, ArH), 8.11 (d, 2H, *J* = 8.6 Hz, ArH), 7.79–7.74 (m, 2H, ArH), 7.56–7.52 (m, 3H, ArH), 7.41 (t, 1H, *J* = 7.6 Hz, ArH), 7.32 (dd, 1H, *J* = 5.2, 2.0 Hz, ArH), 7.22 (d, 1H, *J* = 7.44 Hz, ArH), 3.87 (s, 3H, OCH_3_), 2.40 (s, 3H, CCH_3_). ^13^C-NMR (151 MHz, DMSO-*d*_6_) δ164.82, 164.72, 153.22, 151.92, 149.74, 147.53, 138.24, 134.19, 130.13, 128.99, 125.93, 122.57, 122.28, 119.78, 115.51, 112.89, 52.67, 21.14. HRMS Calcd for C_22_H_18_N_4_O_3_ [M]: 386.1379; found for C_22_H_18_N_4_O_3_ [M]: 386.1370.

*Methyl 4-(4-(4-(3-fluorophenyl)-1H-1,2,3-triazol-1-yl)phenoxy)picolinate* (**8s**). Yield: 96%, white solid. m.p.: 154–157 °C. ^1^H-NMR (600 MHz, CDCl_3_) δ 9.42 (s, 1H, triazole-H), 8.65 (d, 1H, *J* = 5.5 Hz, ArH), 8.08 (d, 2H, *J* = 8.6 Hz, ArH), 7.81 (d, 1H, *J* = 7.6 Hz, ArH), 7.75 (d, 1H, *J* = 9.9 Hz, ArH), 7.58–7.53 (m, 4H, ArH), 7.32 (dd, 1H, *J* = 5.2, 2.0 Hz, ArH), 7.25 (t, 1H, *J* = 7.1 Hz, ArH), 3.86 (s, 3H, CH_3_). ^13^C-NMR (150 MHz, DMSO-*d*_6_) δ 164.84, 164.70, 163.47, 161.86, 153.39, 151.96, 149.76, 146.34, 134.07, 132.62, 132.56, 131.35, 131.29, 122.46, 122.34, 121.46, 120.70, 115.56, 115.17, 115.03, 112.93, 112.06, 111.91, 52.70. HRMS Calcd for C_21_H_15_FN_4_O_3_ [M]: 390.1128; found for C_21_H_15_FN_4_O_3_ [M]: 390.1142.

*Methyl 4-(4-(4-(4-chlorophenyl)-1H-1,2,3-triazol-1-yl)phenoxy)picolinate* (**8t**). Yield: 99%, white solid. m.p.: 173–175 °C. IR (KCl, cm^−1^): 3415.6, 1717.4, 1631.5, 1608.1, 1586.3, 884.0, 866.4, 855.6, 835.6. ^1^H-NMR (600 MHz, DMSO-*d*_6_) δ 9.40 (s, 1H, triazole-H), 8.65 (d, 1H, *J* = 5.6 Hz, ArH), 8.09 (d, 2H, *J* = 8.7 Hz, ArH), 7.98 (d, 2H, *J* = 8.3 Hz, ArH), 7.60 (d, 2H, *J* = 8.2 Hz, ArH), 7.56–7.53 (m, 3H, ArH), 7.32 (dd, 1H, *J* = 5.3, 1.7 Hz, ArH), 3.86 (s, 3H, CH_3_). ^13^C-NMR (151 MHz, DMSO-*d*_6_) δ 164.82, 164.69, 153.34, 151.93, 149.75, 146.35, 134.09, 132.80, 129.18, 129.14, 127.07, 122.42, 122.30, 120.28, 115.53, 112.90, 52.67. HRMS Calcd for C_21_H_15_ClN_4_O_3_ [M]: 406.0833; found for C_21_H_15_ClN_4_O_3_ [M]: 406.0827.

*Methyl 4-(4-(4-(2-(trifluoromethyl)phenyl)-1H-1,2,3-triazol-1-yl)phenoxy)picolinate* (**8u**). Yield: 99%, white solid. m.p.: 87–88 °C. ^1^H-NMR (600 MHz, DMSO-*d_6_*) δ 9.06 (s, 1H, triazole-H), 8.65 (d, 1H, *J* = 5.5 Hz, ArH), 8.14 (d, 2H, *J* = 8.8 Hz, ArH), 7.93 (d, 1H, *J* = 7.9 Hz, ArH), 7.83–7.82 (m, 2H, ArH), 7.72 (t, 1H, *J* = 6.8 Hz, ArH), 7.55–7.52 (m, 3H, ArH), 7.32 (dd, 1H, *J* = 5.5, 2.4 Hz, ArH), 3.86 (s, 3H, CH_3_). ^13^C-NMR (151 MHz, DMSO-*d*_6_) δ 164.81, 164.71, 153.39, 151.92, 149.75, 144.88, 133.93, 132.78, 132.25, 129.39, 128.98, 127.11 (q, *J* = 30.0 Hz), 126.53 (q, *J* = 5.4 Hz), 124.91, 123.10, 122.58, 122.33, 115.51, 112.89, 52.67. HRMS Calcd for C_22_H_15_F_3_N_4_O_3_ [M]: 440.1096; found for C_22_H_15_F_3_N_4_O_3_ [M]: 440.1062.

*Methyl 4-(4-(4-(3-(trifluoromethyl)phenyl)-1H-1,2,3-triazol-1-yl)phenoxy)picolinate* (**8v**). Yield: 93%, white solid. m.p.: 172–174 °C. ^1^H-NMR (600 MHz, CDCl_3_) δ 9.55 (s, 1H, triazole-H), 8.65 (d, 1H, *J* = 5.6 Hz, ArH), 8.28 (d, 2H, *J* = 5.0 Hz, ArH), 8.11 (dt, 2H, *J* = 8.9, 3.3 Hz, ArH), 7.77–7.76 (m, 2H, ArH), 7.55–7.53 (m, 3H, ArH), 7.32 (dd, 1H, *J* = 5.6, 2.6 Hz, ArH), 3.86 (s, 3H, CH_3_). ^13^C-NMR (151 MHz, DMSO-*d*_6_) δ 164.78, 164.64, 153.39, 151.90, 149.73, 145.97, 134.00, 131.29, 130.33, 130.21 (q, *J* = 31.9 Hz), 129.08, 126.85, 125.05, 124.82, 124.80, 123.24, 122.34, 122.28, 121.69, 121.67, 121.44, 120.90, 120.88, 115.51, 112.90, 52.63. HRMS Calcd for C_22_H_15_F_3_N_4_O_3_ [M]: 440.1096; found for C_22_H_15_F_3_N_4_O_3_ [M]: 440.1102.

*Methyl 4-(4-(4-(4-chloro-3-methylphenyl)-1H-1,2,3-triazol-1-yl)phenoxy)picolinate* (**8w**). Yield: 99%, white solid. m.p.: 177–178 °C. ^1^H-NMR (600 MHz, DMSO-*d*_6_) δ 9.38 (s, 1H, triazole-H), 8.65 (d, 1H, *J* = 5.5 Hz, ArH), 8.10 (dt, 2H, *J* = 6.8, 3.3 Hz, ArH), 7.95 (d, 1H, *J* = 1.5 Hz, ArH), 7.80 (dd, 1H, *J* = 8.2, 1.8 Hz, ArH), 7.57–7.53 (m, 4H, ArH), 7.32 (dd, 1H, *J* = 5.6, 2.6 Hz, ArH), 3.86 (s, 3H, OCH_3_), 2.42 (s, 3H, CCH_3_). ^13^C-NMR (151 MHz, DMSO-*d*_6_) δ 164.84, 164.71, 153.33, 151.95, 149.76, 146.48, 136.23, 134.11, 133.06, 129.62, 129.16, 127.97, 124.55, 122.37, 122.32, 120.17, 115.55, 112.93, 52.70, 19.78. HRMS Calcd for C_22_H_17_ClN_4_O_3_ [M]: 420.0989; found for C_22_H_17_ClN_4_O_3_ [M]: 420.1007.

*Methyl 4-(4-(4-(4-chloro-3-(trifluoromethyl)phenyl)-1H-1,2,3-triazol-1-yl)phenoxy)picolinate* (**8x**). Yield: 99%, white solid. m.p.: 175–177 °C. ^1^H-NMR (600 MHz, DMSO-*d*_6_) δ 9.59 (s, 1H, triazole-H), 8.66 (d, 1H, *J* = 5.6 Hz, ArH), 8.38 (d, 1H, *J* = 1.8 Hz, ArH), 8.27 (dd, 1H, *J* = 8.3, 1.9 Hz, ArH), 8.09 (dt, 2H, *J* = 8.9, 1.9 Hz, ArH), 7.92 (d, 1H, *J* = 8.4 Hz, ArH), 7.56–7.54 (m, 3H, ArH), 7.33 (dd, 1H, *J* = 5.5, 2.5 Hz, ArH), 3.87 (s, 3H, CH_3_). ^13^C-NMR (151 MHz, DMSO-*d*_6_) δ 164.81, 164.64, 153.47, 151.93, 149.75, 145.09, 133.93, 132.62, 130.46, 130.12, 129.92, 127.52 (q, *J* = 30.8 Hz), 124.27, 123.71, 122.39, 122.31, 121.90, 121.20, 115.55, 112.94, 52.67. HRMS Calcd for C_22_H_14_ClF_3_N_4_O_3_ [M]: 474.0707; found for C_22_H_14_ClF_3_N_4_O_3_ [M]: 474.0730.

*Methyl 4-(4-(4-(thiophen-2-yl)-1H-1,2,3-triazol-1-yl)phenoxy)picolinate* (**8y**). Yield: 99%, white solid. m.p.: 152–153 °C. ^1^H-NMR (600 MHz, DMSO-*d*_6_) δ 9.26 (s, 1H, triazole-H), 8.65 (d, 1H, *J* = 5.5 Hz, ArH), 8.09 (d, 2H, *J* = 8.9 Hz, ArH), 7.63 (d, 1H, *J* = 5.0 Hz, ArH), 7.56–7.52 (m, 4H, ArH), 7.32 (dd, 1H, *J* = 5.5, 2.5 Hz, ArH), 7.21 (dt, 1H, *J* = 3.7, 1.2 Hz, ArH), 3.87 (s, 3H, CH_3_). ^13^C-NMR (151 MHz, DMSO-*d*_6_) δ 164.82, 164.69, 153.36, 151.93, 149.75, 142.85, 133.99, 132.29, 128.11, 126.06, 124.74, 122.46, 122.27, 119.12, 115.53, 112.91, 52.67. HRMS Calcd for C_19_H_14_N_4_O_3_S [M]: 378.0787; found for C_19_H_14_N_4_O_3_S [M]: 378.0797.

### 3.4. Cell Viability Assay

The viability of HT-29, MCF-7, and HEK293T cells was determined using an MTT (3-(4,5-dimethylthiazol-2-yl)-2,5-diphenyltetrazolium bromide) assay. These cancer cell lines were cultured in minimum essential medium supplemented with 10% foetal bovine serum.

Approximately 4 × 10^3^ cells were suspended in cell culture medium, plated in a 96-well plate and incubated at 37 °C in 5% CO_2_ for 24 h. The test compounds were added to the culture medium and incubated for a further 72 h. Fresh MTT was then added to each well at a final concentration of 5 μg/mL, and incubated with the cells at 37 °C for 4 h. The formazan crystals in each well were dissolved in 100 μL DMSO, and the absorbance at 492 nm (MTT formazan wavelength) and 630 nm (reference wavelength) was measured by a microplate reader. The reported IC_50_ results represented averages of at least three determinations and were calculated using the Bacus Laboratories Incorporated Slide Scanner (Bliss) software.

### 3.5. Colony Formation Assay

Logarithmic phase HT-29 cells were seeded into 6-well plates at a concentration of 500 cells per well. The next day, cells were treated with **8f** at 0.125, 0.25, 0.5, and 1 μM. Colonies were fixed with methanol at room temperature for 15 min and then stained with a solution of 0.1% crystal violet diluted by phosphate buffered saline (PBS) from 0.5% crystal violet (dissolved in methanol) for 15 min and then washed by water. The stained colonies were then photographed and dissolved by 33% acetic acid. Optical density of absorbance was read at 570 nm.

### 3.6. Apoptosis Assay

Apoptosis of HT-29 cells was detected using a flow cytometric assay. Briefly, cells were seeded in 6-well plates and incubated overnight. The following day, cells were treated with different concentrations of compound **8f** for 72 h. The cells and supernatants were harvested and washed twice with cold PBS and then resuspended in 100μL 1× Binding Buffer. 5 µL of FITC Annexin V and 5 µL PI were added in each tube and the cells were then gently vortexed incubated for 15 min at RT (25 °C) in the dark. In addition, 400 µL of 1× Binding Buffer was then added to each tube. The stained cells were analyzed by a flow cytometer (Thermo Fisher Scientific, San Jose, CA, USA).

## 4. Conclusions

In summary, we demonstrated the synthetic application of our highly active CuAAC reaction catalyst in preparing a novel series of sorafenib analogs. The binuclear Cu catalyst exhibited a satisfactory performance in the synthesis of the target compounds. At 0.1–0.3 mol % catalyst loading, all triazole products were obtained at 91–99% isolated yields. Using this catalytic system, 25 new compounds were prepared effectively and were evaluated biologically. The MTT and colony formation assays demonstrated that compound **8f** potently inhibited the proliferation of HT-29 cells. The flow cytometric assay suggested that **8f** caused significant apoptosis of HT-29 cells in a dose-dependent manner.

According to the chemical structural characteristics of **8f**, we considered it to be a kinase inhibitor. Actually, we attempted to identify its biological target by screening it against a panel of kinases including B-Raf, VEGFR-3, PDGFRα, fibroblast growth factor receptor 1 (FGFR1), c-Kit, TRAF2 and NCK interacting kinase (TNIK), ret proto-oncogene (RET), cyclin-dependent 2 (CDK2), checkpoint kinase 1 (CHK1), and cyclin-dependent kinase 4 (CDK4). However, none of these kinases were significantly inhibited (Supplementary materials Table S1). These results indicated that 8f acted quite differently from sorafenib. More detailed investigations of the mechanisms of action of these compounds are in progress in our laboratory. Studies of the SARs indicated that a fluorine atom at the meta-position of the terminal phenyl ring was well tolerated, rather than the 3-CF_3_-4-Cl group in sorafenib. The hydrogen-bond donor at the 2-position of pyridine was indispensable for the activity of this chemical series. These findings all provide potentially valuable guidance for designing more powerful compounds.

## Figures and Tables

**Table 1:** Antiproliferative activities and calculated logP-values of compounds **8a**–**8y**.

Products	IC_50_ (μM) ^a^		ClogP ^b^
	HT-29	MCF-7	
**8a**	0.74 ± 0.12	7.53 ± 0.65	3.45
**8b**	8.53 ± 1.09	10.17 ± 0.77	3.97
**8c**	>100	>100	3.97
**8d**	89.83 ± 3.28	>100	3.30
**8e**	0.84 ± 0.17	0.33 ± 0.02	3.60
**8f**	0.20 ± 0.01	0.61 ± 0.12	3.60
**8g**	>100	>100	4.06
**8h**	>100	>100	4.22
**8i**	0.55 ± 0.08	10.99 ± 1.66	4.33
**8j**	0.76 ± 0.13	26.70 ± 3.37	4.33
**8k**	13.51 ± 1.28	50.96 ± 2.76	4.57
**8l**	49.20 ± 2.39	99.19 ± 3.58	4.94
**8m**	0.60 ± 0.05	7.21± 0.96	2.62
**8n**	0.58 ± 0.13	0.73 ± 0.32	3.23
**8o**	>100	>100	0.74
**8p**	>100	>100	1.74
**8q**	15.22 ± 2.68	71.92 ± 3.66	4.18
**8r**	5.33 ± 0.31	48.33 ± 2.56	4.70
**8s**	9.12 ± 0.60	17.06 ± 2.17	4.33
**8t**	11.81 ± 2.31	36.23 ± 1.70	4.79
**8u**	44.54 ± 2.28	43.93 ± 2.67	5.06
**8v**	>100	>100	5.06
**8w**	9.57 ± 1.05	44.08 ± 2.69	5.30
**8x**	54.98 ± 2.70	40.84 ± 3.56	5.67
**8y**	8.79 ± 0.97	47.36 ± 1.38	3.96
**sorafenib**	5.29 ± 0.32	43.30 ± 1.36	4.34

^a^ Biological data are generated from at least three independent experiments; ^b^ ClogP-values calculated using MarvinSketch 6.1.0.

## Supplementary Materials

Supplementary materials are available online. Table S1: Enzymatic screening of selected compounds.
