# Supplementary material for: Synthesis and Antitumor Activity of Triazole-Containing Sorafenib Analogs
Source: Molecules. 2017 Oct 24;22(10):1759. doi: 10.3390/molecules22101759 (PMC6151694; doi:10.3390/molecules22101759)

Supplementary information

Synthesis and antitumor activity of triazole–containing sorafenib analogs

Wenjing Ye, Qi Yao, Simiao Yu, Ping Gong, and Mingze Qin*

Key Laboratory of Structure-Based Drug Design and Discovery (Shenyang Pharmaceutical University), Ministry of Education, Shenyang 110016, PR China

Table S1. Enzymatic screening of selected compounds.

Compound	% inhibition at 10 μM[a]									
	VEGFR-3	PDGFRα	FGFR1	c-KIT	CDK4	RET	TNIK	B-Raf	CDK2	CHK1
**8e**	1.2	3.4	15	7.3	-2.6	28.9	18.6	5.6	17	21
**8f**	3.5	-5.2	1.8	-2.6	-2.3	17.2	15.1	5.6	8.4	10
**8j**	4.3	0.6	-0.61	11	0.7	18.0	13.1	5.6	13	17
**8n**	15	-0.5	8.0	9.5	2.3	11.0	26.6	13.8	8.9	10

[a] The biological data are generated from at least two independent experiments.

Copies of IR spectra for triazoles

8e

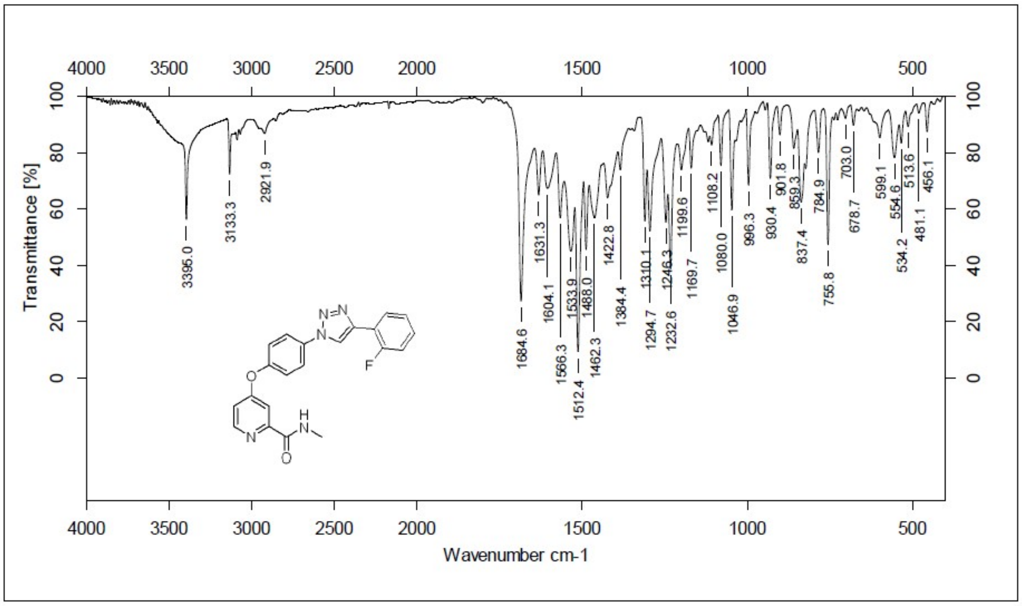

Sample : KPR-6	Frequency Range : 3999.64 - 400.157		Measured on : 2017-10-11
Technique : Sample form	Resolution : 2	Instrument : EQUINOX55	Sample Scans : 16
Customer : Default	Zerofilling : 2	Acquisition : Double Sided,Forv	

8f

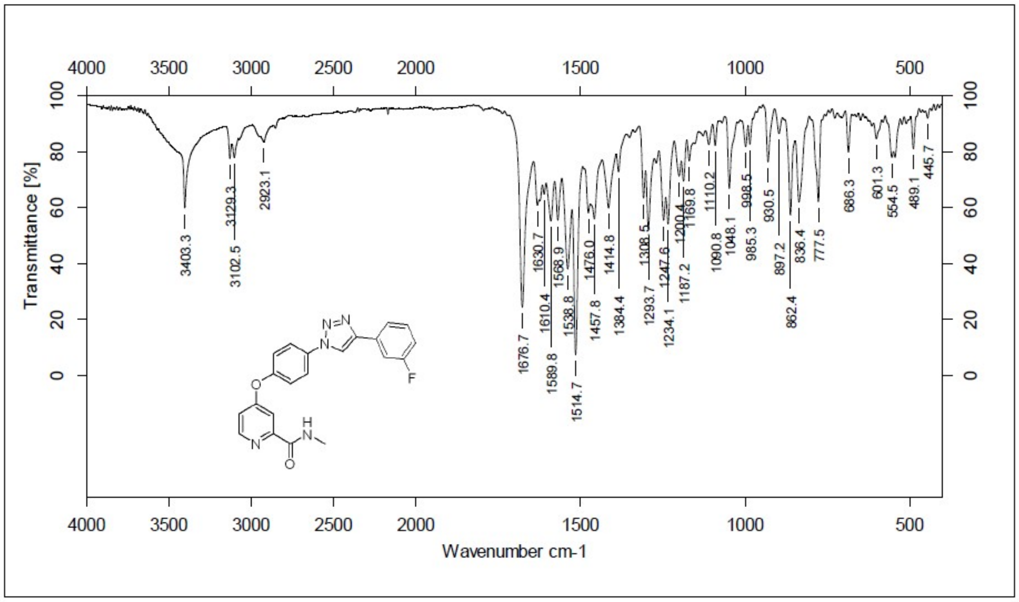

Sample : KPR-2	Frequency Range : 3999.64 - 400.157		Measured on : 2017-10-11
Technique : Sample form	Resolution : 2	Instrument : EQUINOX55	Sample Scans : 16
Customer : Default	Zerofilling : 2	Acquisition : Double Sided,Forv	

8g

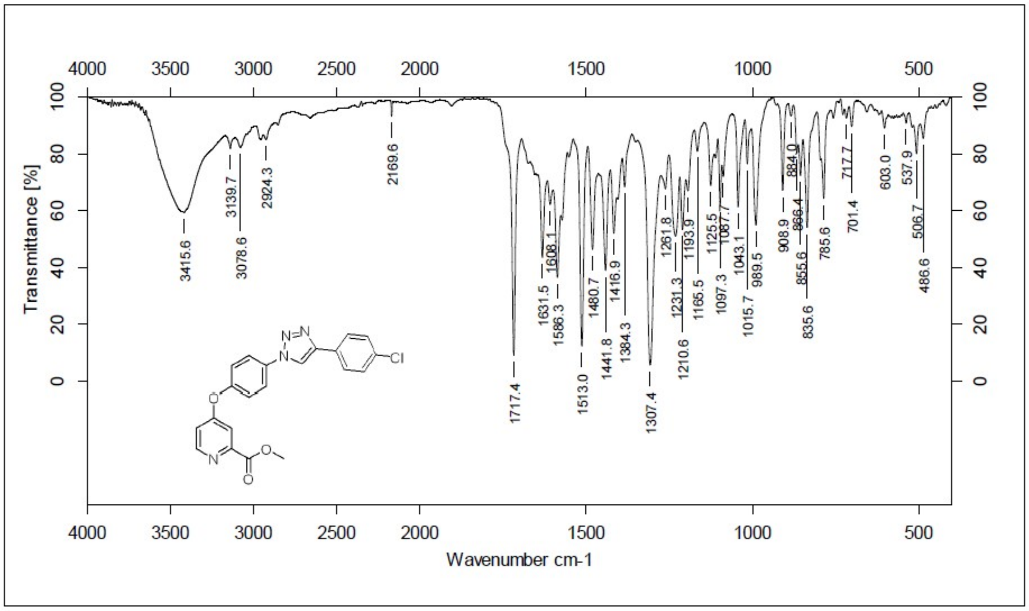

Sample : KPR-28	Frequency Range : 3999.64 - 400.157	Measured on : 2017-10-11	
Technique : Sample form	Resolution : 2	Instrument : EQUINOX55	Sample Scans : 16
Customer : Default	Zerofilling : 2	Acquisition : Double Sided,Forv	

8j

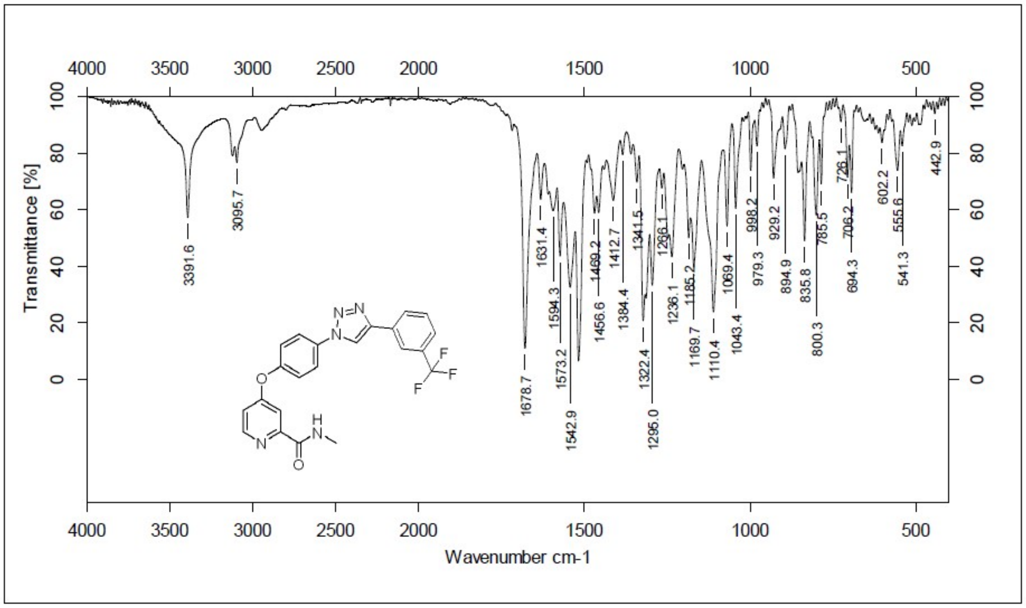

Sample : KPR-35	Frequency Range : 3999.64 - 400.157	Measured on : 2017-10-11	
Technique : Sample form	Resolution : 2	Instrument : EQUINOX55	Sample Scans : 16
Customer : Default	Zerofilling : 2	Acquisition : Double Sided,Forv	

8n

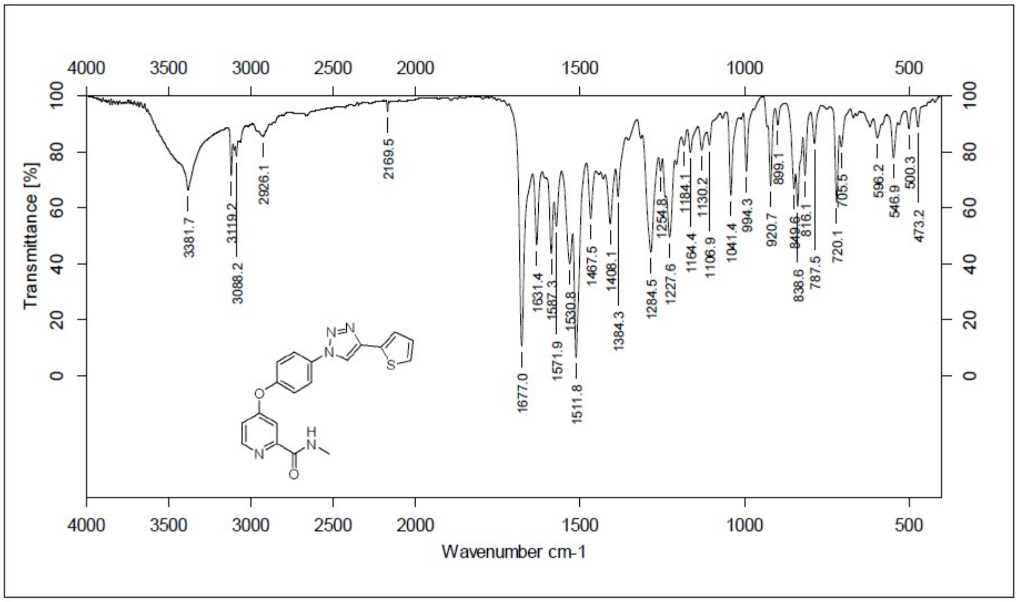

Sample : KPR-4		Frequency Range : 3999.64 - 400.157	Measured on : 2017-10-11
Technique : Sample form	Resolution : 2	Instrument : EQUINOX55	Sample Scans : 16
Customer : Default	Zerofilling : 2	Acquisition : Double Sided,Forv	

Copies of NMR spectra for triazoles

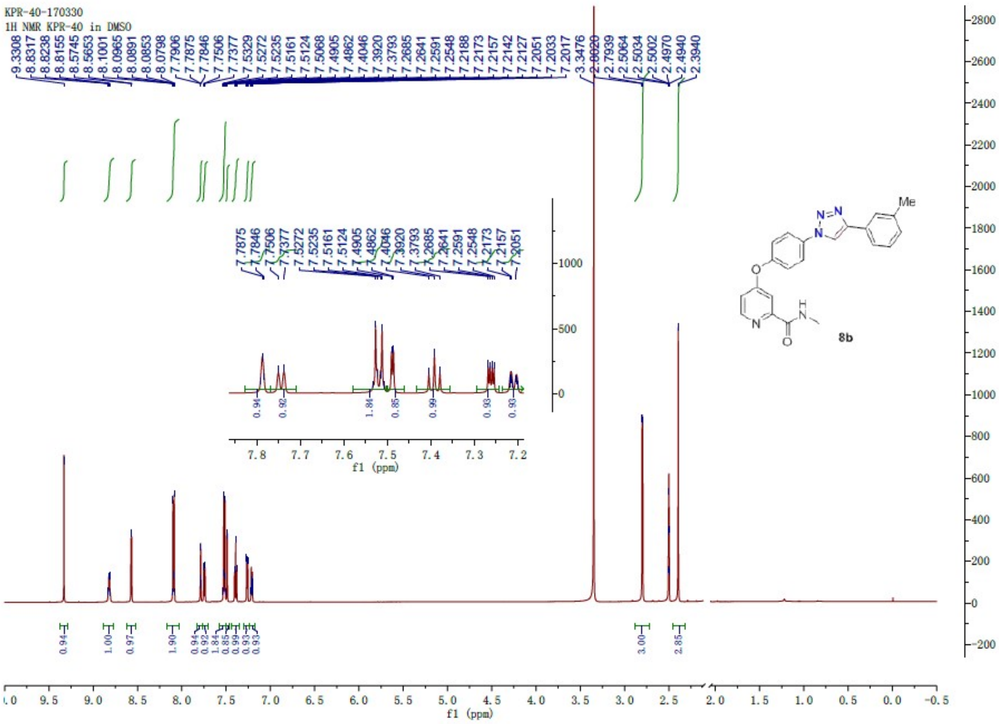

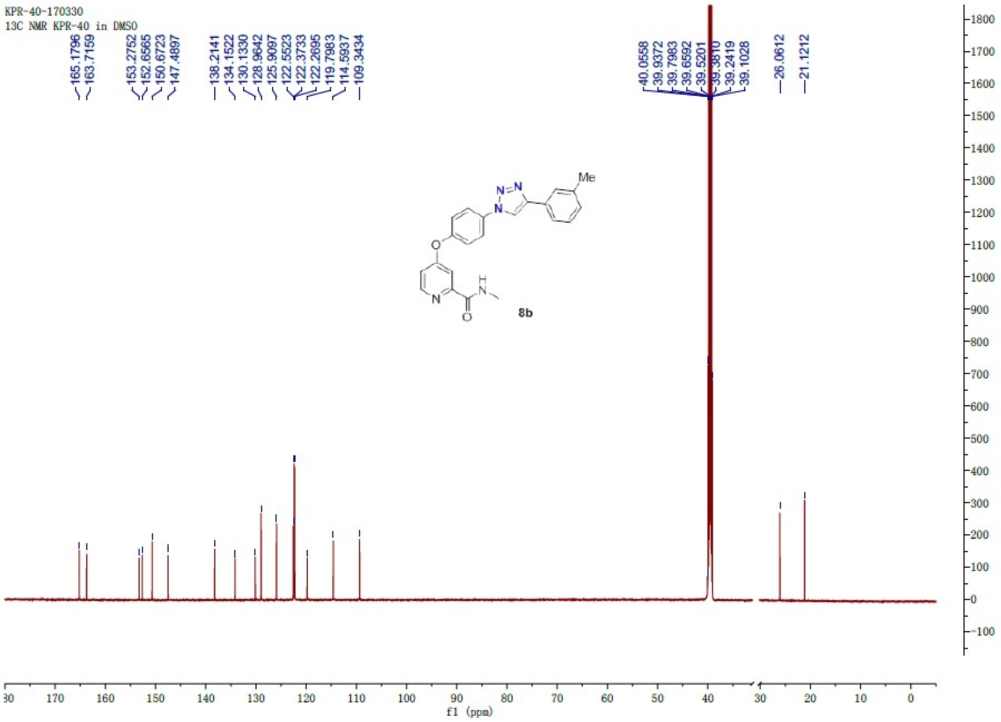
KPR-40-170330
13C NMR KPR-40 in DMSO
165.1796
163.7159
153.2752
152.6565
150.6723
147.4897
138.2141
134.1522
130.1330
128.9642
125.9097
122.5523
122.3733
122.2695
119.7983
114.5937
109.3434
40.0558
39.9372
39.7983
39.6592
39.5201
39.3810
39.2419
39.1028
26.0612
21.1212
Me
8b
f1 (ppm)

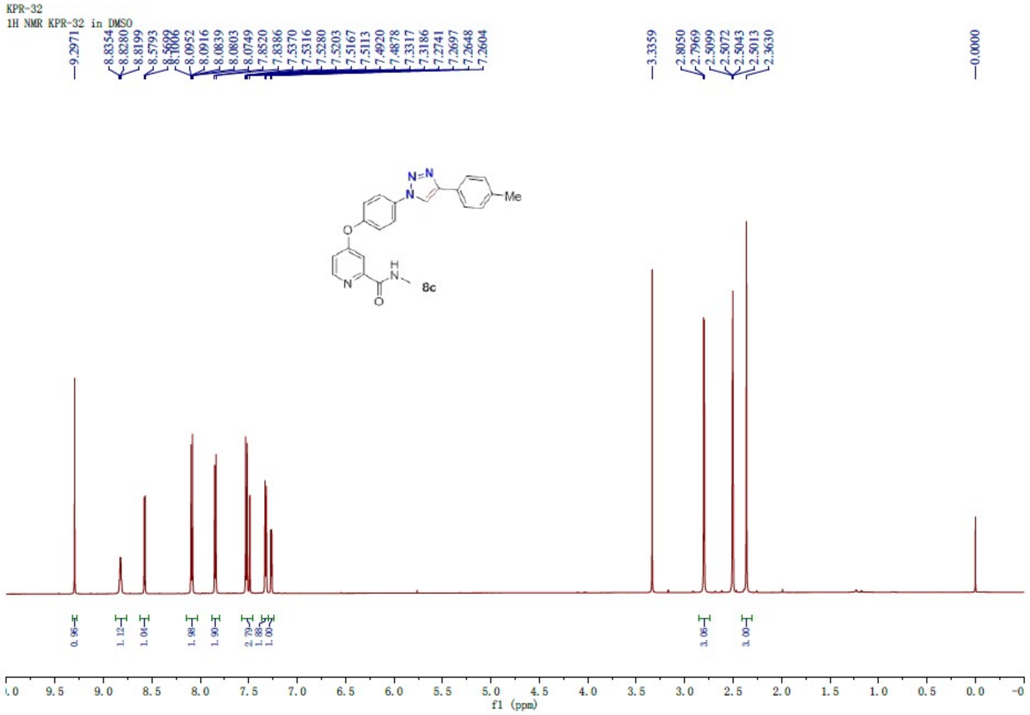
KPR-32
1H NMR KPR-32 in DMSO
9.2971
8.8354
8.8280
8.8199
8.5793
8.5699
8.1006
8.0952
8.0916
8.0839
8.0803
8.0749
7.8520
7.8386
7.5370
7.5316
7.5280
7.5203
7.5167
7.5113
7.4920
7.4878
7.3317
7.3186
7.2741
7.2697
7.2648
7.2604
3.3359
2.8050
2.7969
2.5099
2.5072
2.5043
2.5013
2.3630
0.0000
Me
8c
f1 (ppm)

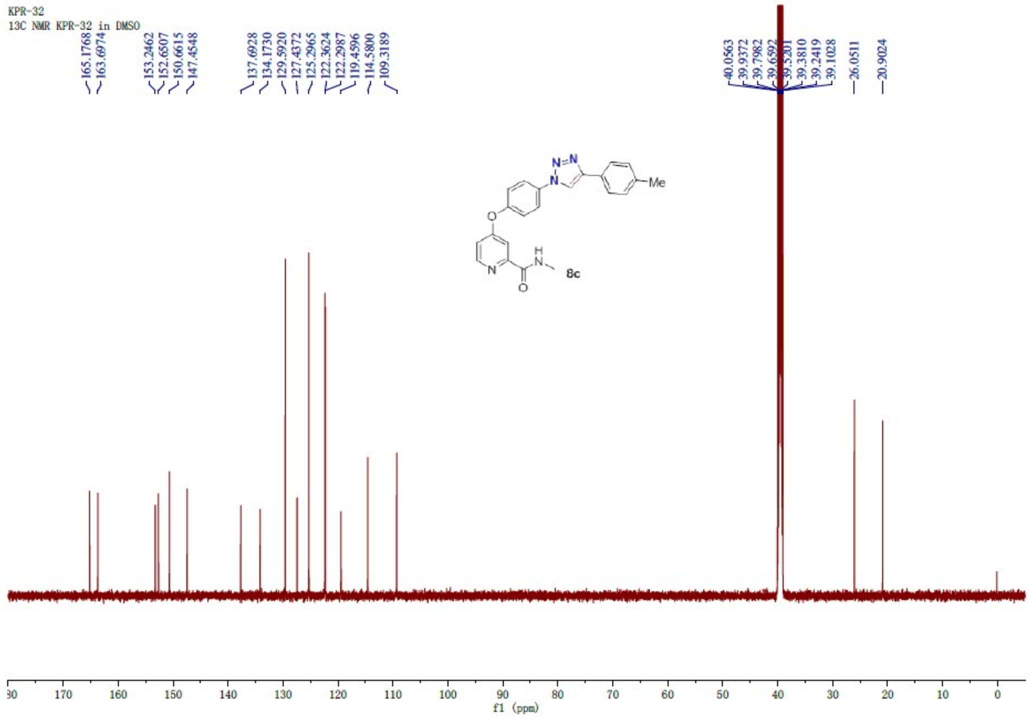
KPR-32
13C NMR KPR-32 in DMSO
165.1768
163.6974
153.2462
152.6507
150.6615
147.4548
137.6928
134.1730
129.5920
127.4372
125.2965
122.3624
122.2987
119.4596
114.5800
109.3189
40.0563
39.9372
39.7982
39.5201
39.3810
39.2419
39.1028
26.0511
20.9024
Me
N
N
N
O
N
H
O
8c
f1 (ppm)

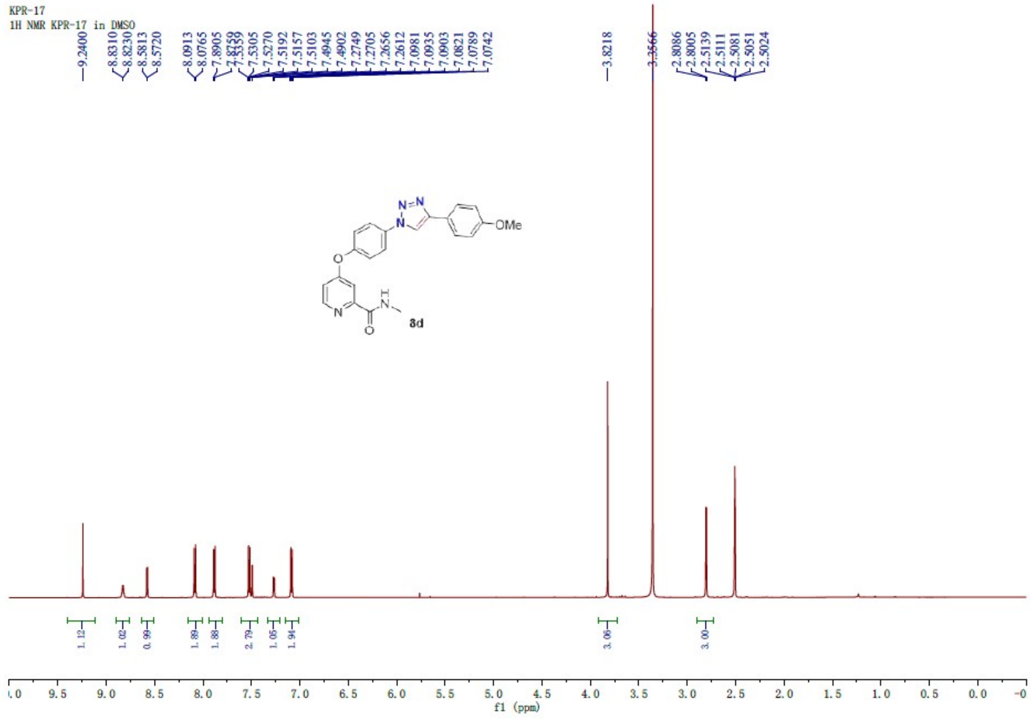
KPR-17
1H NMR KPR-17 in DMSO
9.2400
8.8310
8.8230
8.5813
8.5720
8.0913
8.0765
7.8905
7.5305
7.5270
7.5192
7.5157
7.5103
7.4945
7.4902
7.2749
7.2705
7.2656
7.2612
7.0981
7.0935
7.0903
7.0821
7.0789
7.0742
3.8218
3.3566
2.8086
2.8005
2.5139
2.5111
2.5081
2.5051
2.5024
OMe
N
N
N
O
N
H
O
8d
1.12
1.02
0.99
1.89
1.88
2.79
1.05
1.94
3.06
3.00
f1 (ppm)

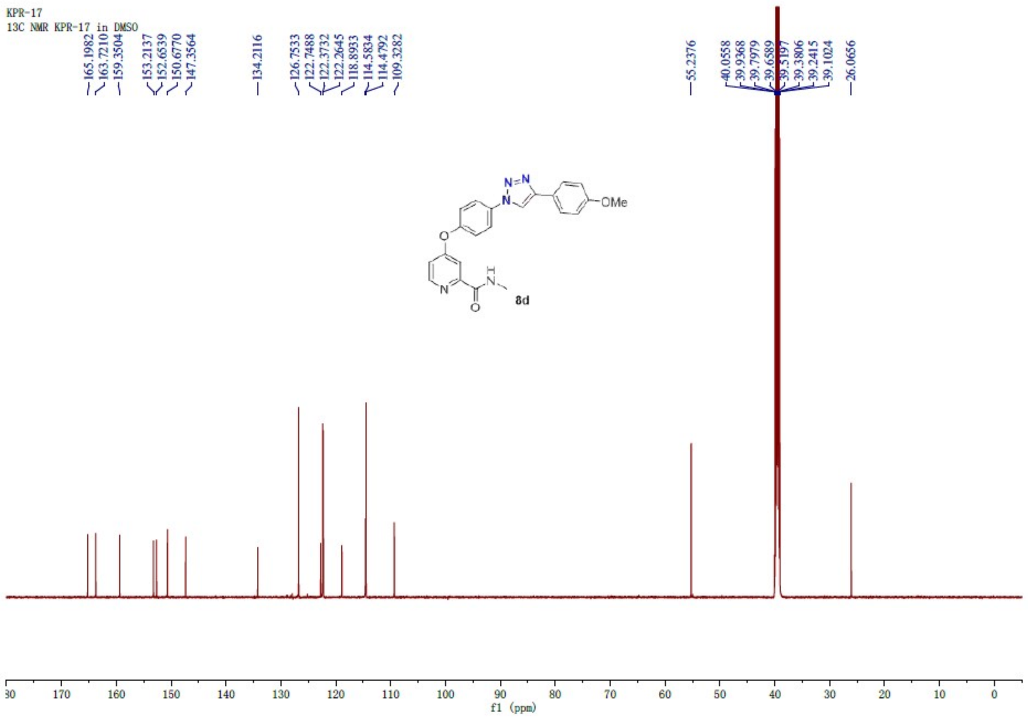
KPR-17
13C NMR KPR-17 in DMSO
165.1982
163.7210
159.3504
153.2137
152.6539
150.6770
147.3564
134.2116
126.7533
122.7488
122.3732
122.2645
118.8933
114.5834
114.4792
109.3282
55.2376
40.0558
39.9368
39.7979
39.6589
39.5197
39.3806
39.2415
39.1024
26.0656
OMe
8d
f1 (ppm)

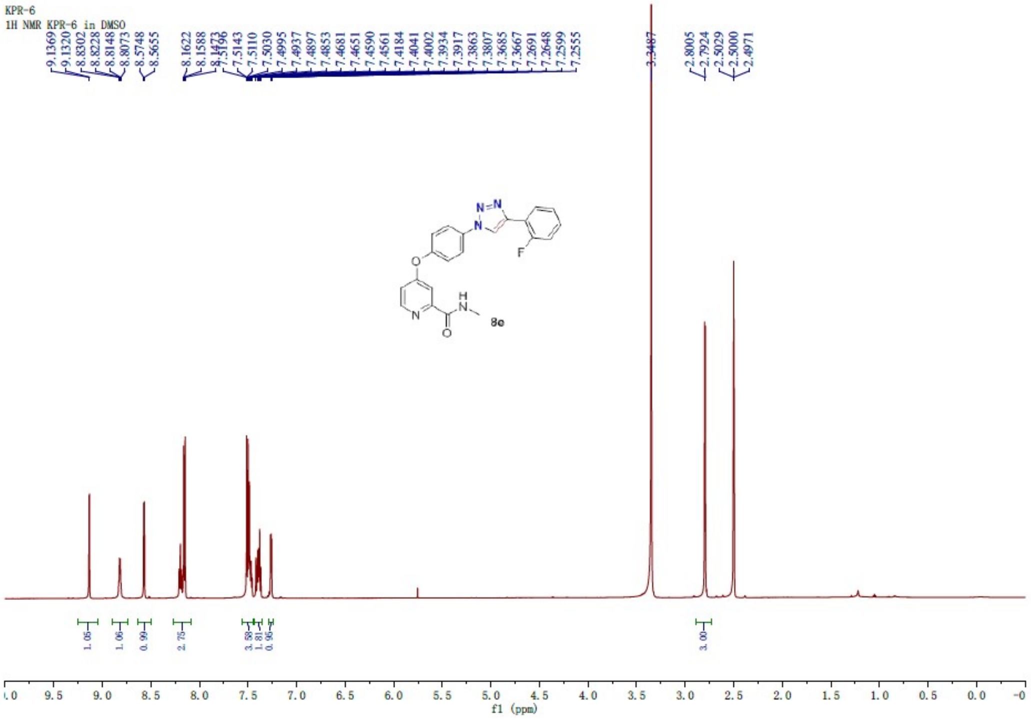
KPR-6
1H NMR KPR-6 in DMSO
9.1369
9.1320
8.8302
8.8228
8.8148
8.8073
8.5748
8.5655
8.1622
8.1588
7.5143
7.5110
7.5030
7.4995
7.4937
7.4897
7.4853
7.4681
7.4651
7.4590
7.4561
7.4184
7.4041
7.4002
7.3934
7.3917
7.3863
7.3807
7.3685
7.3667
7.2691
7.2648
7.2599
7.2555
3.3487
2.8005
2.7924
2.5029
2.5000
2.4971
F
8e
1.06
1.06
0.99
2.75
3.58
1.81
0.96
3.00
f1 (ppm)

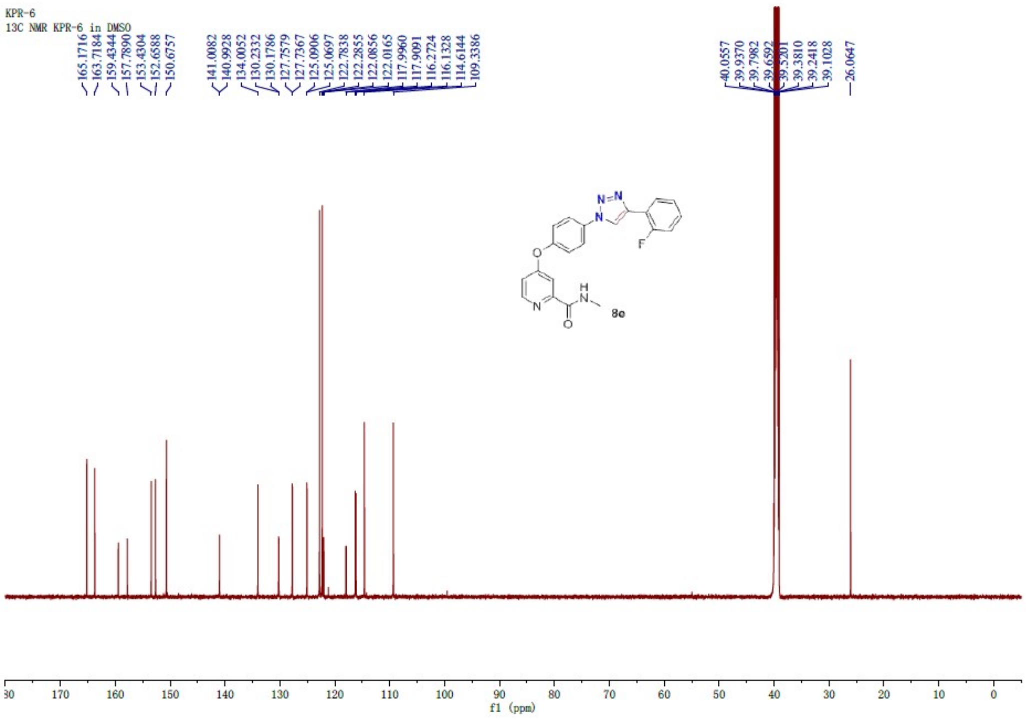
KPR-6
13C NMR KPR-6 in DMSO
165.1716
163.7184
159.4344
157.7890
153.4304
152.6588
150.6757
141.0082
140.9928
134.0052
130.2332
130.1786
127.7579
127.7367
125.0906
125.0697
122.7838
122.2855
122.0856
122.0165
117.9960
117.9091
116.2724
116.1328
114.6144
109.3386
40.0557
39.9370
39.7982
39.6592
39.5201
39.3810
39.2418
39.1028
26.0647
8e
f1 (ppm)

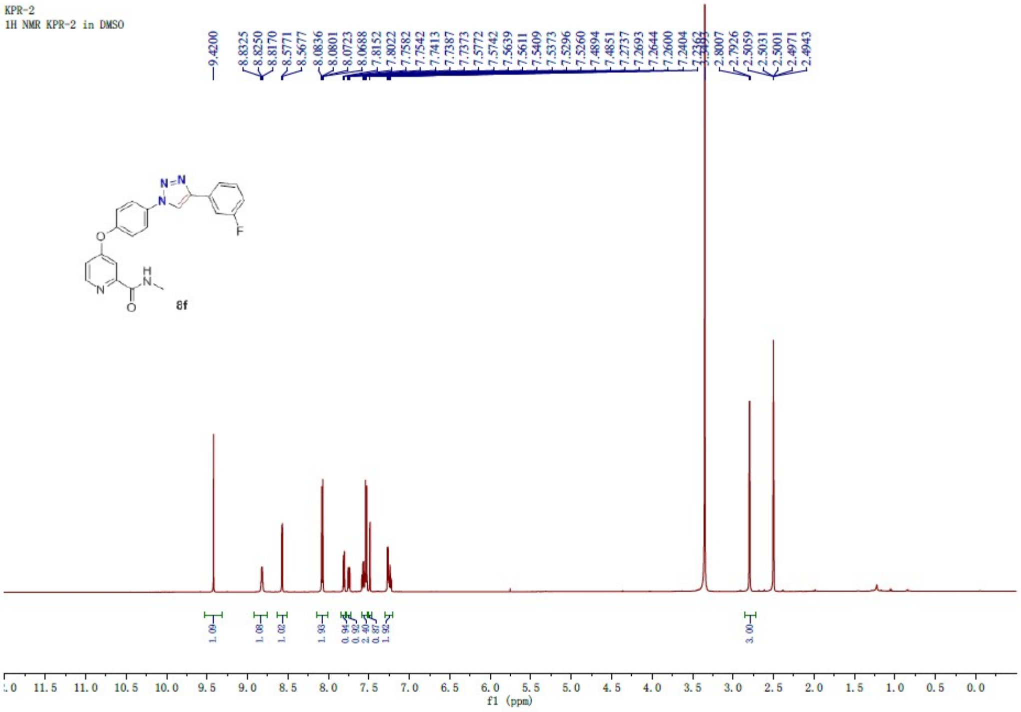
KPR-2
1H NMR KPR-2 in DMSO
9.4200
8.8325
8.8250
8.8170
8.5771
8.5677
8.0836
8.0801
8.0723
8.0688
7.8152
7.8022
7.7582
7.7542
7.7413
7.7387
7.7373
7.5772
7.5742
7.5639
7.5611
7.5409
7.5373
7.5296
7.5260
7.4894
7.4851
7.2737
7.2693
7.2644
7.2600
7.2404
2.8007
2.7926
2.5059
2.5031
2.5001
2.4971
2.4943
8f
f1 (ppm)

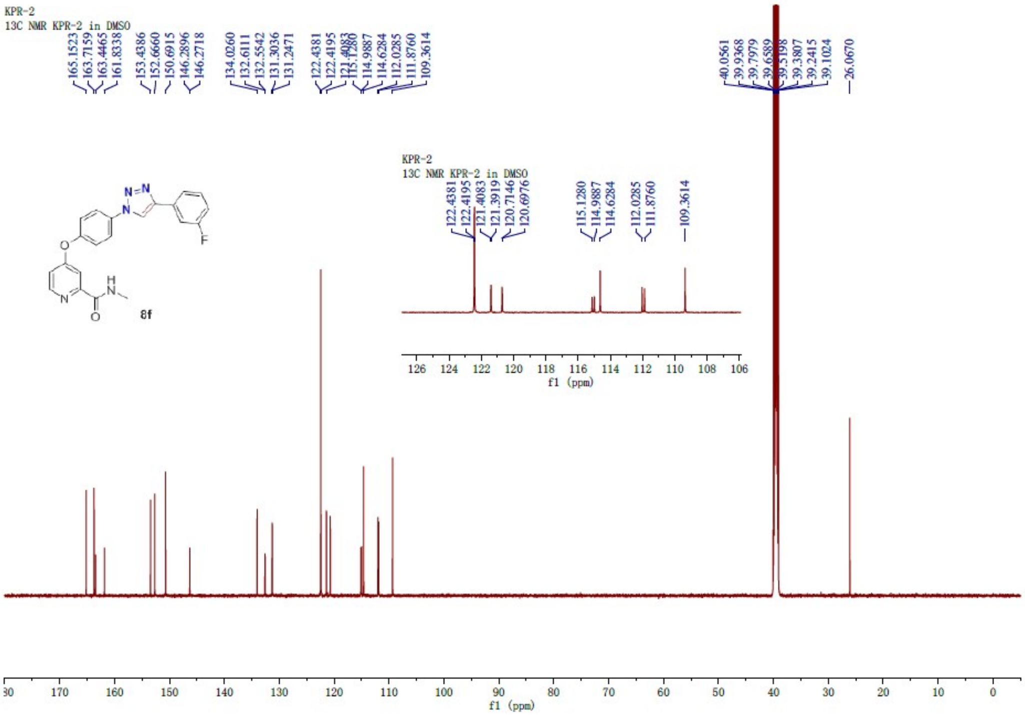
KPR-2
13C NMR KPR-2 in DMSO
165.1523
163.7159
163.4465
161.8338
153.4386
152.6660
150.6915
146.2896
146.2718
134.0260
132.6111
132.5542
131.3036
131.2471
122.4381
122.4195
121.4083
115.1280
114.9887
114.6284
112.0285
111.8760
109.3614
40.0561
39.9368
39.7979
39.6589
39.5198
39.3807
39.2415
39.1024
26.0670
8f
KPR-2
13C NMR KPR-2 in DMSO
122.4381
122.4195
121.4083
121.3919
120.7146
120.6976
115.1280
114.9887
114.6284
112.0285
111.8760
109.3614
f1 (ppm)
f1 (ppm)

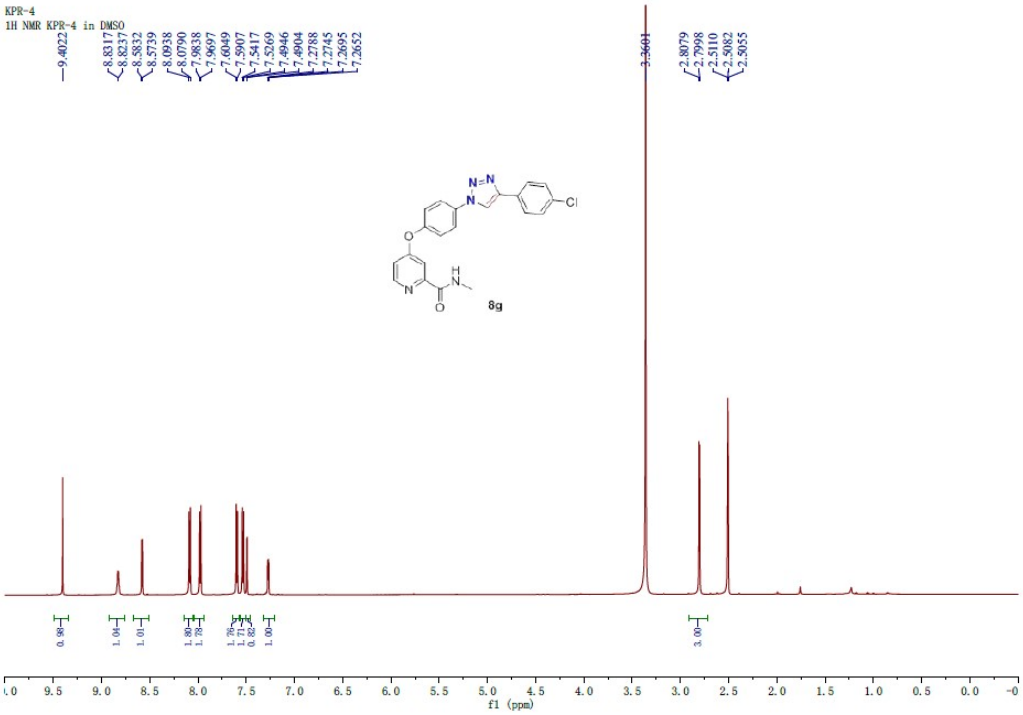
KPR-4
1H NMR KPR-4 in DMSO
9.4022
8.8317
8.8237
8.5832
8.5739
8.0938
8.0790
7.9838
7.9697
7.6049
7.5907
7.5417
7.5269
7.4946
7.4904
7.2788
7.2745
7.2695
7.2652
3.3601
2.8079
2.7998
2.5110
2.5082
2.5055
8g
f1 (ppm)

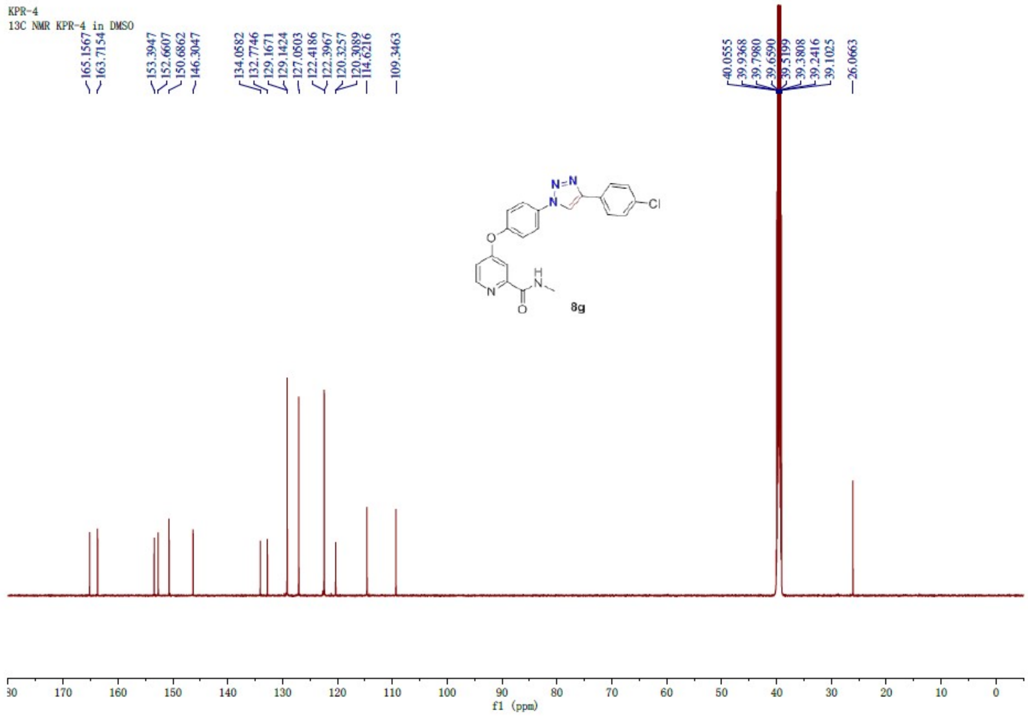
KPR-4
13C NMR KPR-4 in DMSO
165.1567
163.7154
153.3947
152.6607
150.6862
146.3047
134.0582
132.7746
129.1671
129.1424
127.0503
122.4186
122.3967
120.3257
120.3089
114.6216
109.3463
40.0555
39.9368
39.7980
39.6590
39.5199
39.3808
39.2416
39.1025
26.0663
N=N
N
O
N
H
O
Cl
8g
f1 (ppm)

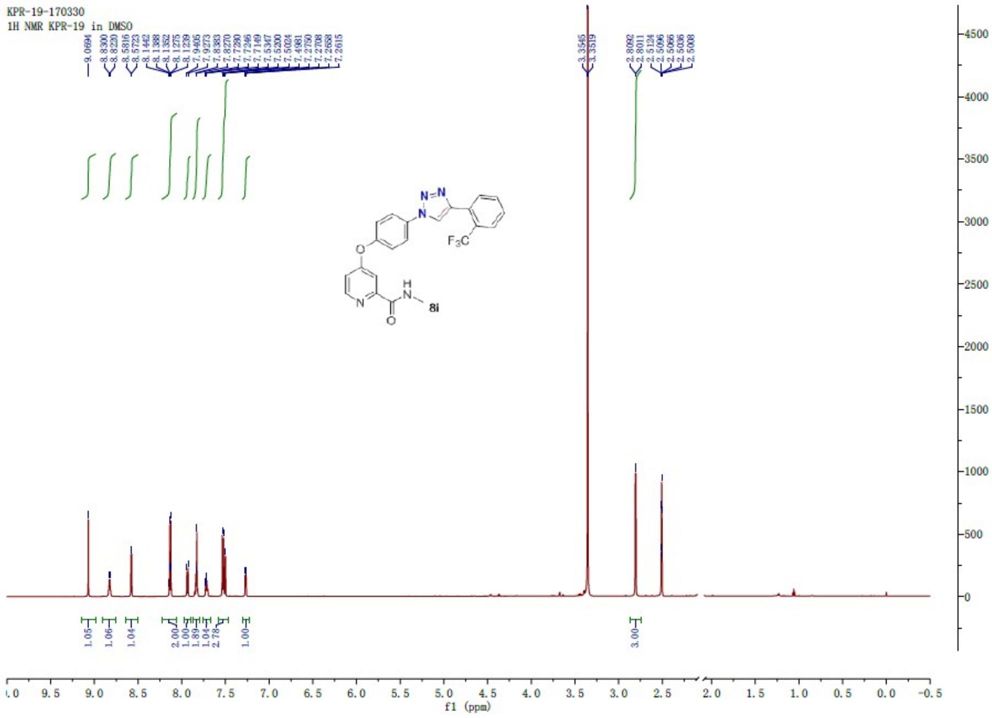
KPR-19-170330
1H NMR KPR-19 in DMSO
9.0694
8.8300
8.8220
8.5816
8.5723
8.1442
8.1388
8.1352
8.1275
8.1239
7.9405
7.9273
7.8383
7.8270
7.7290
7.7246
7.7149
7.5347
7.5200
7.5024
7.4981
7.2750
7.2708
7.2658
7.2615
3.3545
3.3519
2.8092
2.8011
2.5124
2.5096
2.5066
2.5036
2.5008
N=N
N
O
N
H
O
F3C
8i
1.05
1.06
1.04
2.00
1.00
1.89
1.04
2.78
1.00
3.00
f1 (ppm)

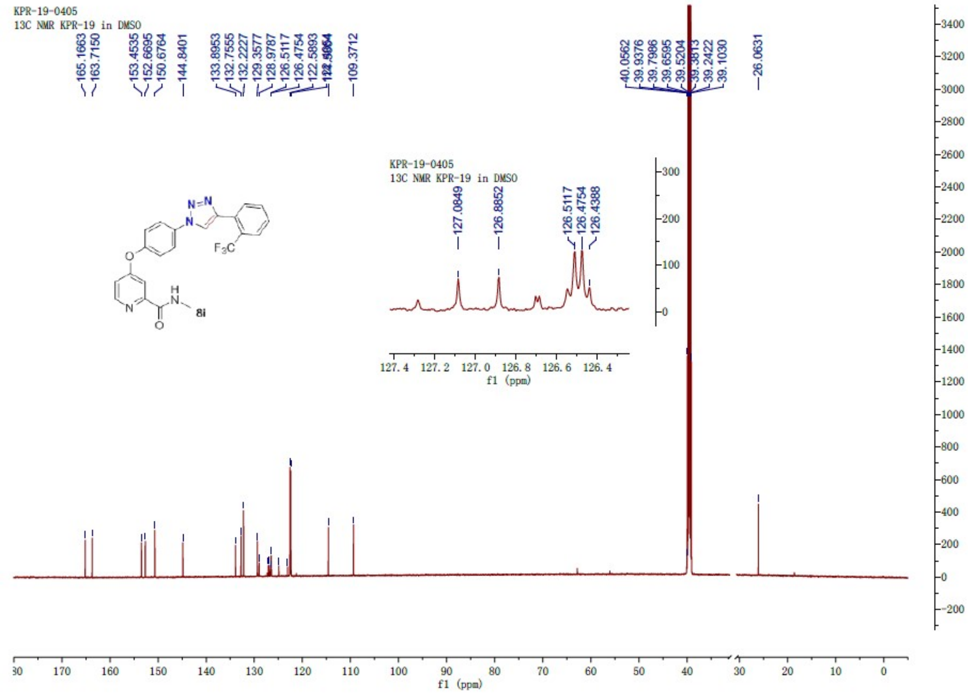
KPR-19-0405
13C NMR KPR-19 in DMSO
165.1663
163.7150
153.4535
152.6695
150.6764
144.8401
133.8953
132.7555
132.2227
129.3577
128.9787
126.5117
126.4754
122.5893
109.3712
40.0562
39.9376
39.7986
39.6595
39.5204
39.3813
39.2422
39.1030
26.0631
8i
127.0849
126.8852
126.5117
126.4754
126.4388
f1 (ppm)

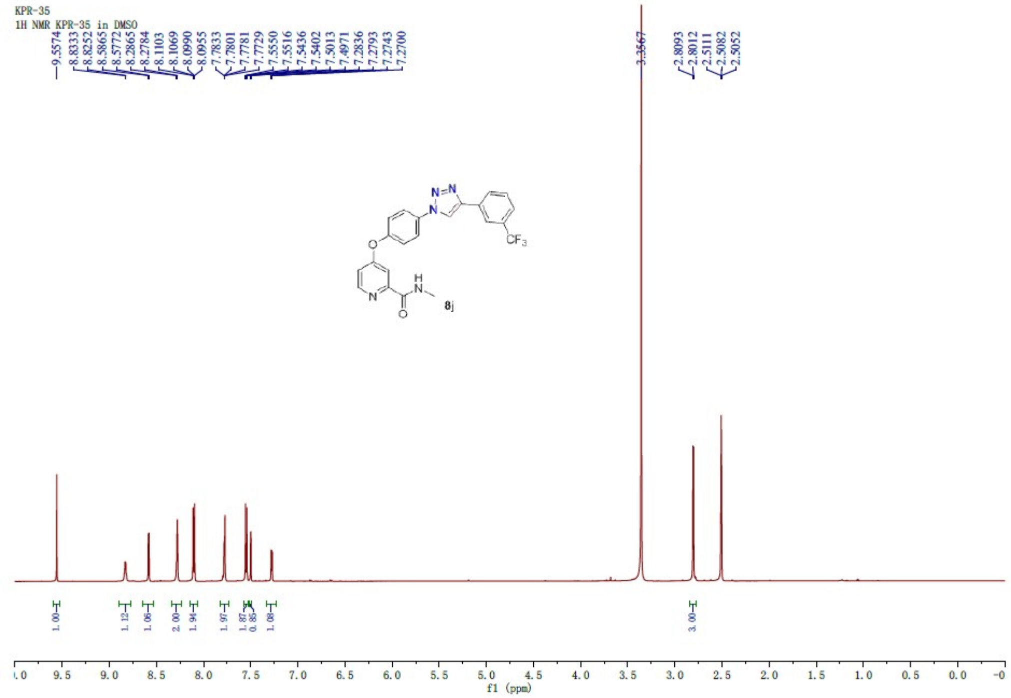
KPR-35
1H NMR KPR-35 in DMSO
9.5574
8.8333
8.8252
8.5865
8.5772
8.2865
8.2784
8.1103
8.1069
8.0990
8.0955
7.7833
7.7801
7.7781
7.7729
7.5550
7.5516
7.5436
7.5402
7.5013
7.4971
7.2836
7.2793
7.2743
7.2700
3.3567
2.8093
2.8012
2.5111
2.5082
2.5052
8j
f1 (ppm)

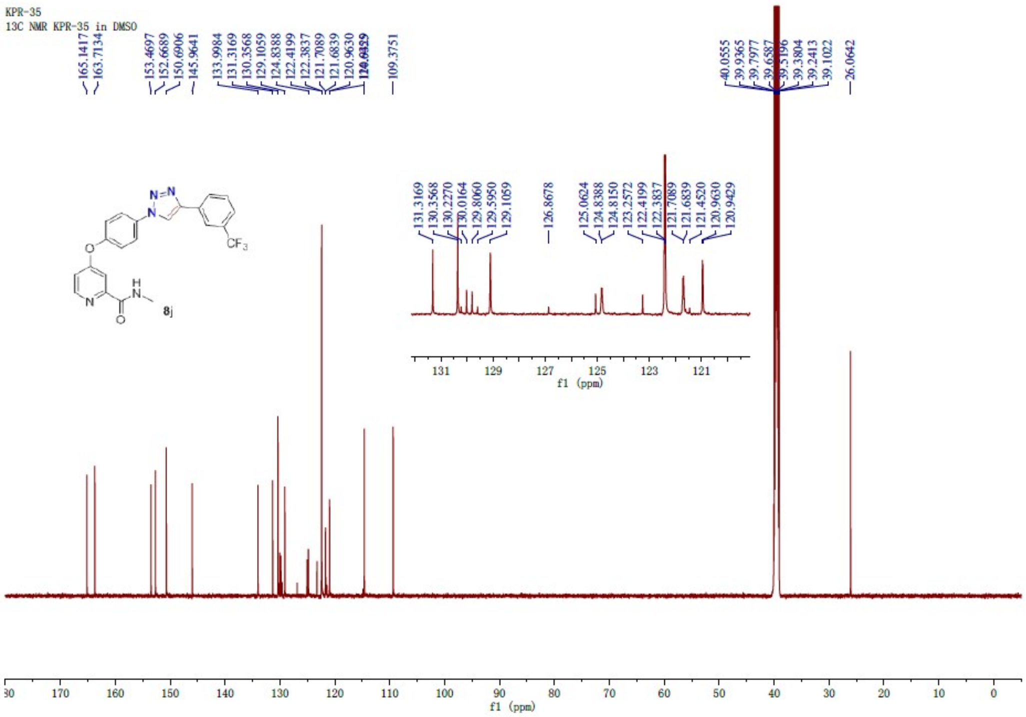
KPR-35
13C NMR KPR-35 in DMSO
165.1417
163.7134
153.4697
152.6689
150.6906
145.9641
133.9984
131.3169
130.3568
129.1059
124.8388
122.4199
122.3837
121.7089
121.6839
120.9630
109.3751
40.0555
39.9365
39.7977
39.5196
39.3804
39.2413
39.1022
26.0642
131.3169
130.3568
130.2270
130.0164
129.8060
129.5950
129.1059
126.8678
125.0624
124.8388
124.8150
123.2572
122.4199
122.3837
121.7089
121.6839
121.4520
120.9630
120.9429
8j
f1 (ppm)

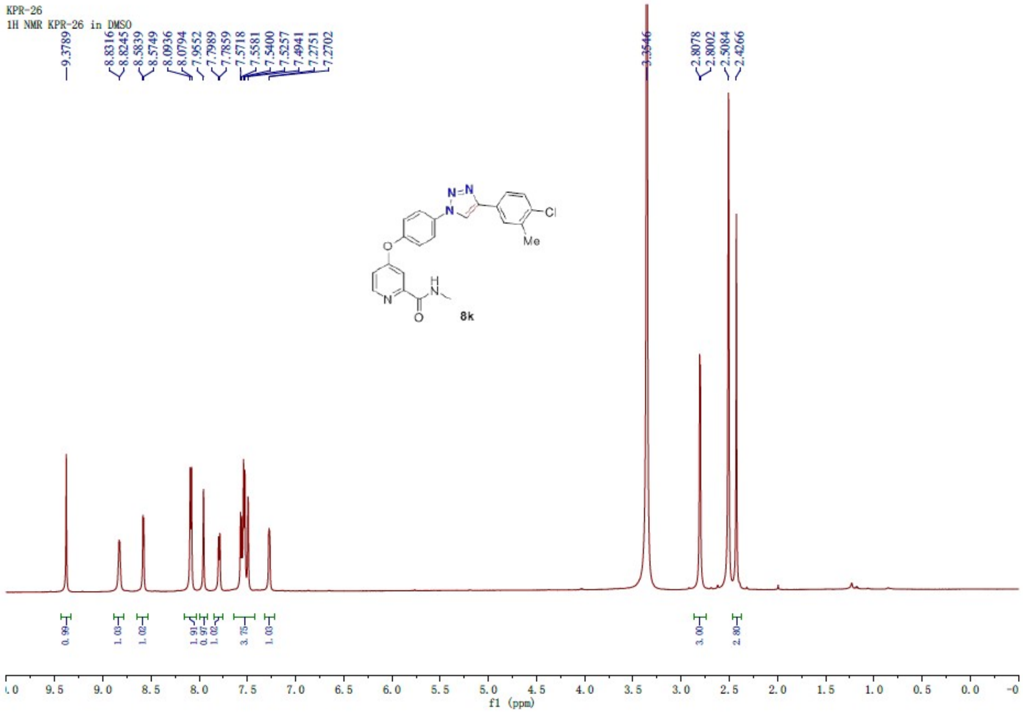
KPR-26
1H NMR KPR-26 in DMSO
9.3789
8.8316
8.8245
8.5839
8.5749
8.0936
8.0794
7.9552
7.7989
7.7859
7.5718
7.5581
7.5400
7.5257
7.4941
7.2751
7.2702
3.3546
2.8078
2.8002
2.5084
2.4266
Cl
Me
8k
f1 (ppm)

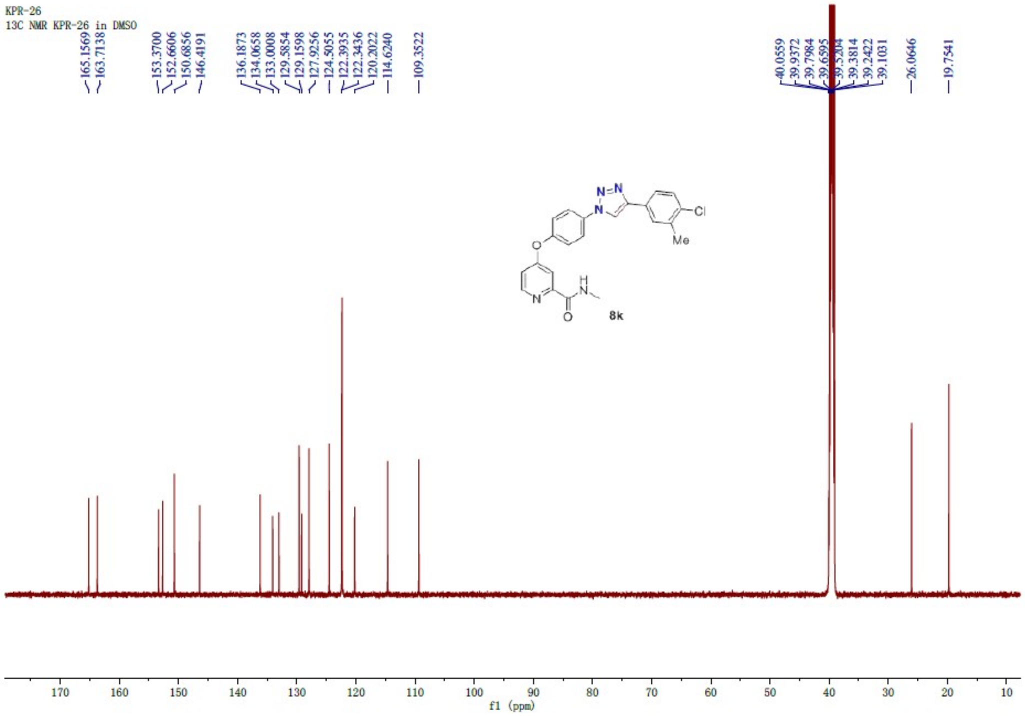
KPR-26
13C NMR KPR-26 in DMSO
165.1569
163.7138
153.3700
152.6606
150.6856
146.4191
136.1873
134.0658
133.0008
129.5854
129.1598
127.9256
124.5055
122.3935
122.3436
120.2022
114.6240
109.3522
40.0559
39.9372
39.7984
39.6595
39.5204
39.3814
39.2422
39.1031
26.0646
19.7541
Cl
Me
N
O
8k
f1 (ppm)

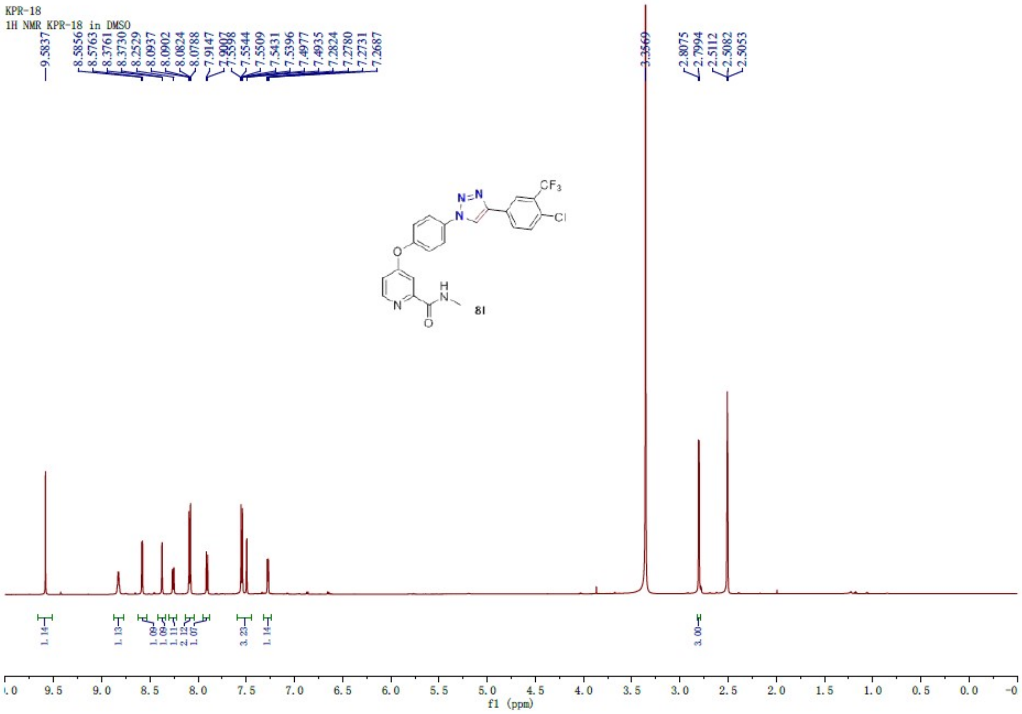
KPR-18
1H NMR KPR-18 in DMSO
9.5837
8.5856
8.5763
8.3761
8.3730
8.2529
8.0937
8.0902
8.0824
8.0788
7.9147
7.5544
7.5509
7.5431
7.5396
7.4977
7.4935
7.2824
7.2780
7.2731
7.2687
3.3569
2.8075
2.7994
2.5112
2.5082
2.5053
CF3
Cl
N
O
8l
1.14
1.13
1.09
1.09
1.11
2.12
1.07
3.23
1.14
3.00
f1 (ppm)

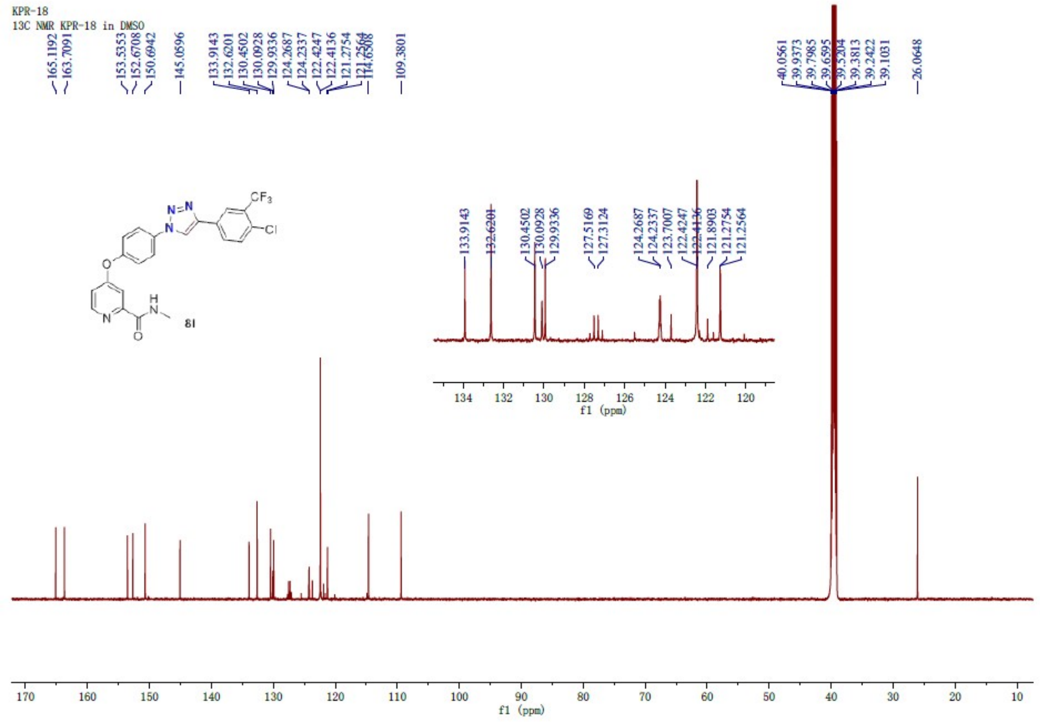
KPR-18
13C NMR KPR-18 in DMSO
8l
f1 (ppm)

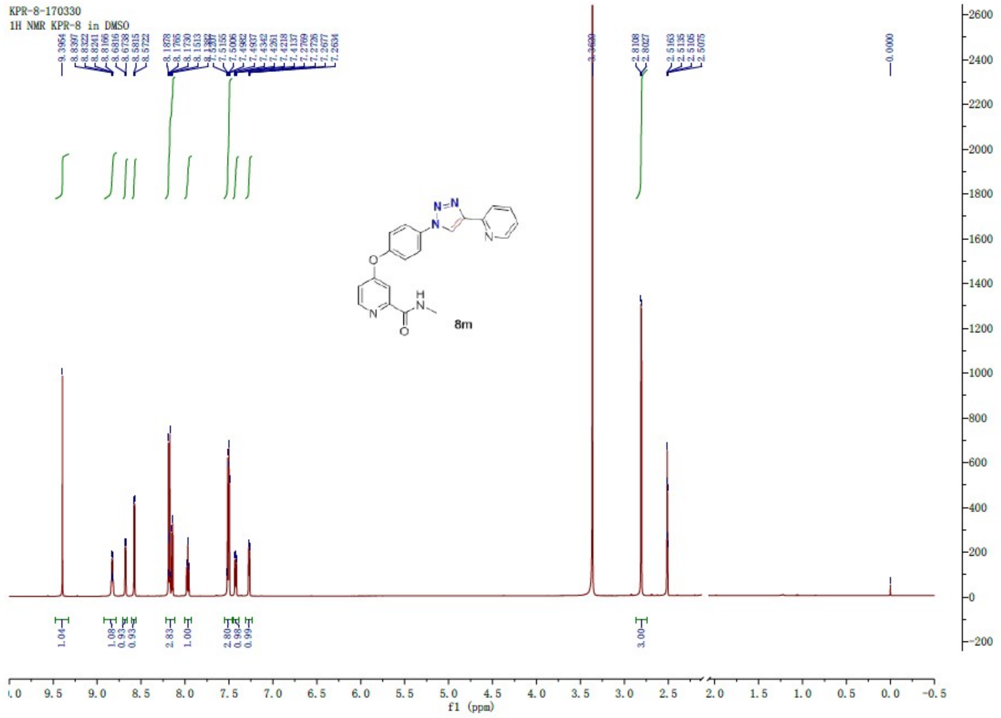
KPR-8-170330
1H NMR KPR-8 in DMSO
8m
f1 (ppm)

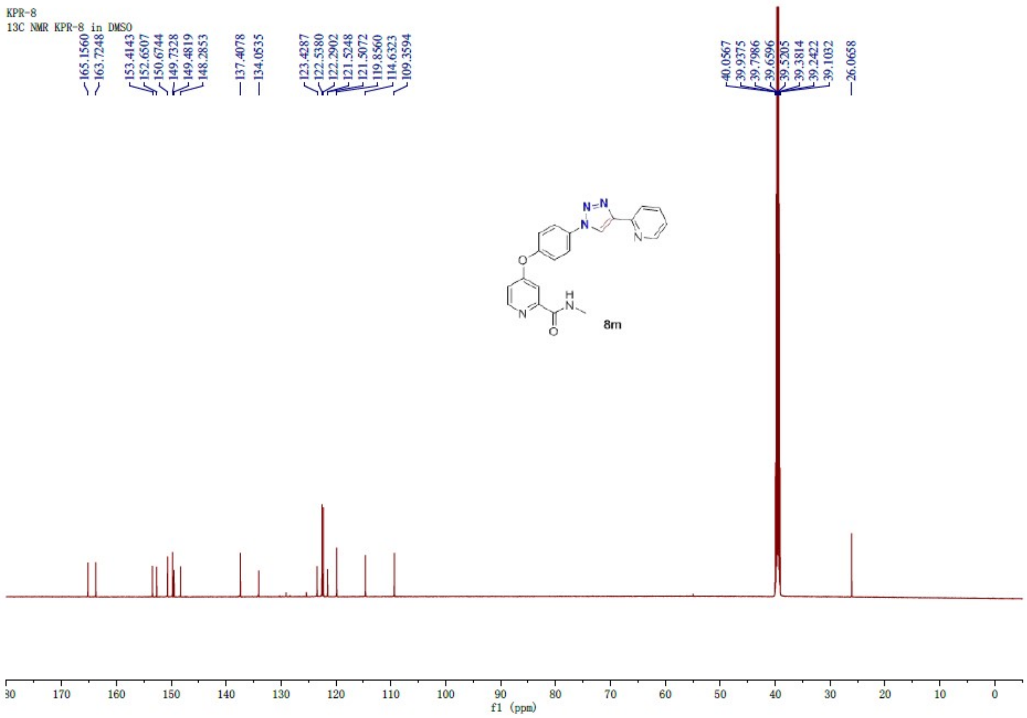
KPR-8
13C NMR KPR-8 in DMSO
165.1560
163.7248
153.4143
152.6507
150.6744
149.7328
149.4819
148.2853
137.4078
134.0535
123.4287
122.5380
122.2902
121.5248
121.5072
119.8560
114.6323
109.3594
40.0567
39.9375
39.7986
39.6596
39.5205
39.3814
39.2422
39.1032
26.0658
8m
f1 (ppm)

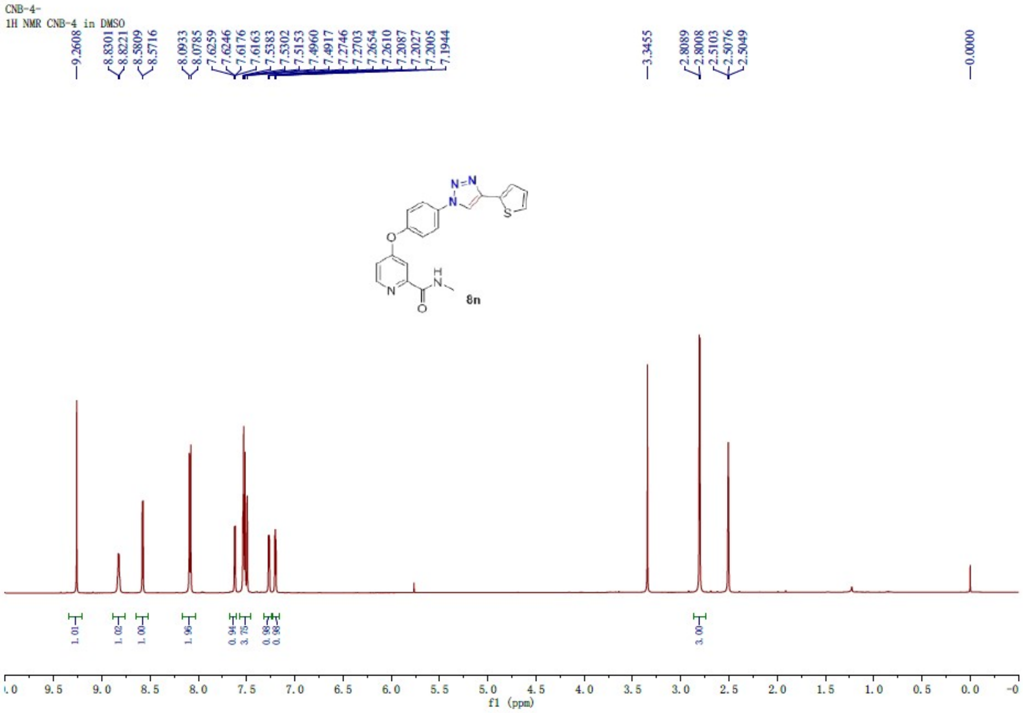
CNB-4-
1H NMR CNB-4 in DMSO
9.2608
8.8301
8.8221
8.5809
8.5716
8.0933
8.0785
7.6259
7.6246
7.6176
7.6163
7.5383
7.5302
7.5153
7.4960
7.4917
7.2746
7.2703
7.2654
7.2610
7.2087
7.2027
7.2005
7.1944
3.3455
2.8089
2.8008
2.5103
2.5076
2.5049
0.0000
8n
1.01
1.02
1.00
1.96
0.94
3.75
0.98
0.98
3.00
f1 (ppm)

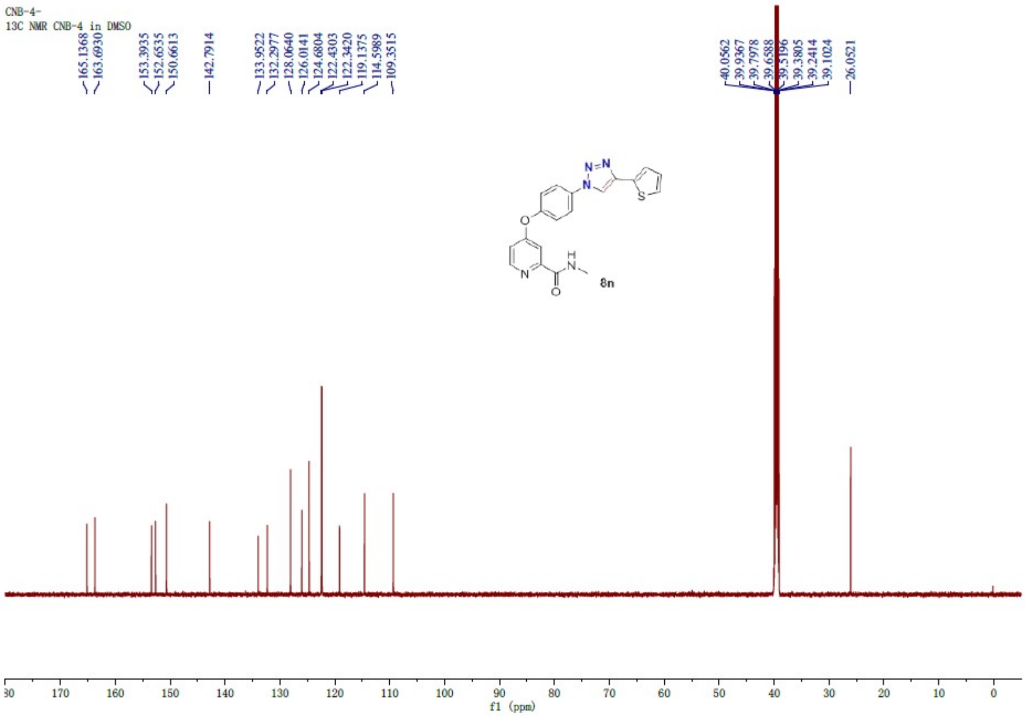
CNB-4-
13C NMR CNB-4 in DMSO
165.1368
163.6930
153.3935
152.6535
150.6613
142.7914
133.9522
132.2977
128.0640
126.0141
124.6804
122.4303
122.3420
119.1375
114.5989
109.3515
40.0562
39.9367
39.7978
39.6588
39.5196
39.3805
39.2414
39.1024
26.0521
8n
f1 (ppm)

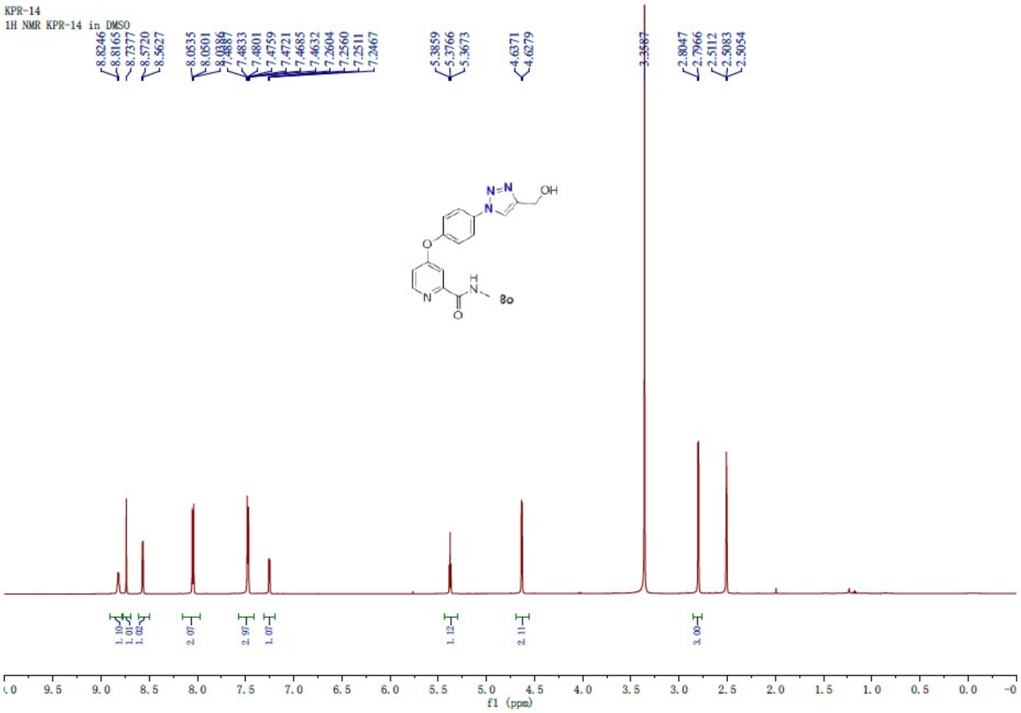
KPR-14
1H NMR KPR-14 in DMSO
8.8246
8.8165
8.7377
8.5720
8.5627
8.0535
8.0501
7.4833
7.4801
7.4759
7.4721
7.4685
7.4632
7.2604
7.2560
7.2511
7.2467
5.3859
5.3766
5.3673
4.6371
4.6279
3.3587
2.8047
2.7966
2.5112
2.5083
2.5054
OH
8o
1.10
1.01
1.02
2.07
2.97
1.07
1.12
2.11
3.00
f1 (ppm)

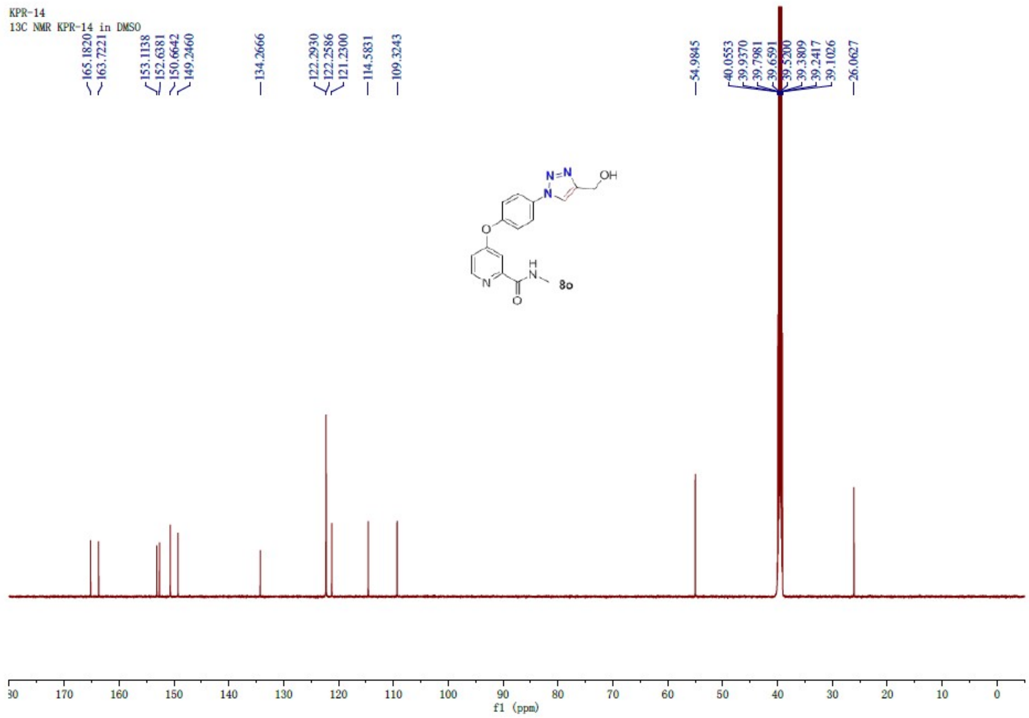
KPR-14
13C NMR KPR-14 in DMSO
165.1820
163.7221
153.1138
152.6381
150.6642
149.2460
134.2666
122.2930
122.2586
121.2300
114.5831
109.3243
54.9845
40.0553
39.9370
39.7981
39.6591
39.5200
39.3809
39.2417
39.1026
26.0627
OH
8o
f1 (ppm)

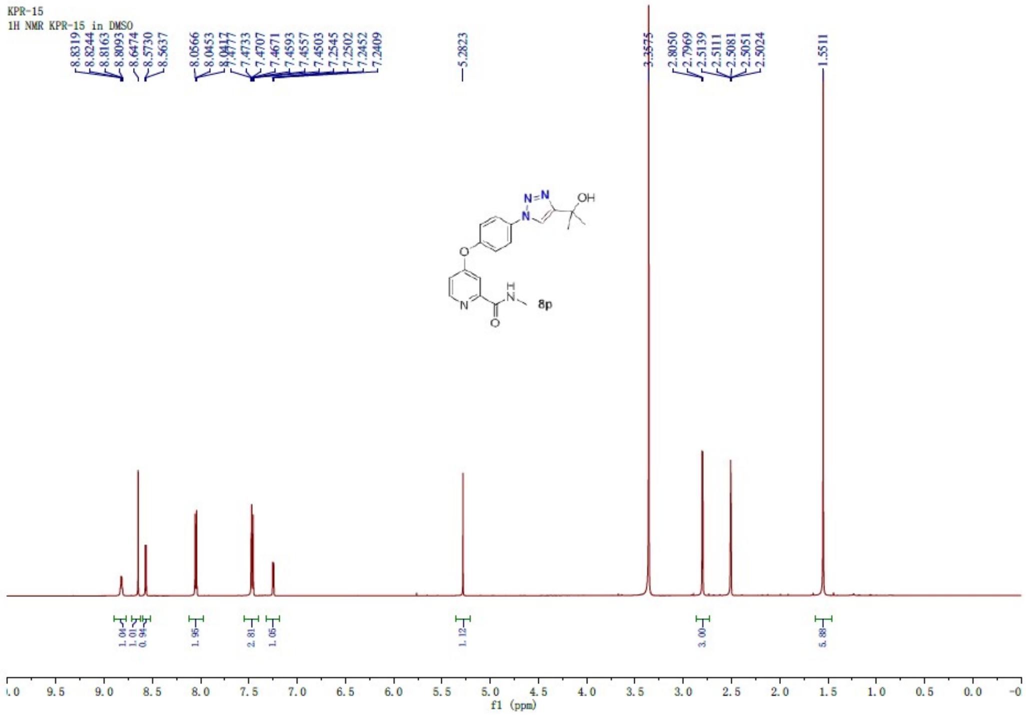
KPR-15
1H NMR KPR-15 in DMSO
8.8319
8.8244
8.8163
8.8093
8.6474
8.5730
8.5637
8.0566
8.0453
7.4733
7.4707
7.4671
7.4593
7.4557
7.4503
7.2545
7.2502
7.2452
7.2409
5.2823
3.3575
2.8050
2.7969
2.5139
2.5111
2.5081
2.5051
2.5024
1.5511
OH
8p
1.04
1.01
0.94
1.96
2.81
1.06
1.12
3.00
5.88
f1 (ppm)

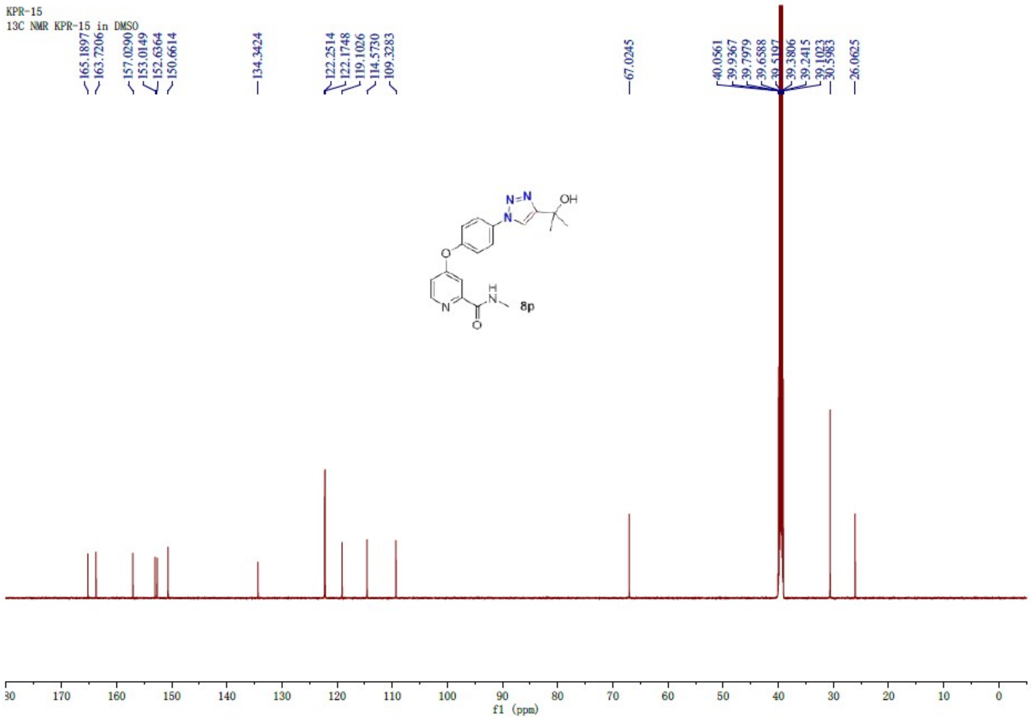
KPR-15
13C NMR KPR-15 in DMSO
165.1897
163.7206
157.0290
153.0149
152.6364
150.6614
134.3424
122.2514
122.1748
119.1026
114.5730
109.3283
67.0245
40.0561
39.9367
39.7979
39.6588
39.5197
39.3806
39.2415
39.1023
30.5983
26.0625
OH
N
N
N
O
N
H
N
O
8p
f1 (ppm)

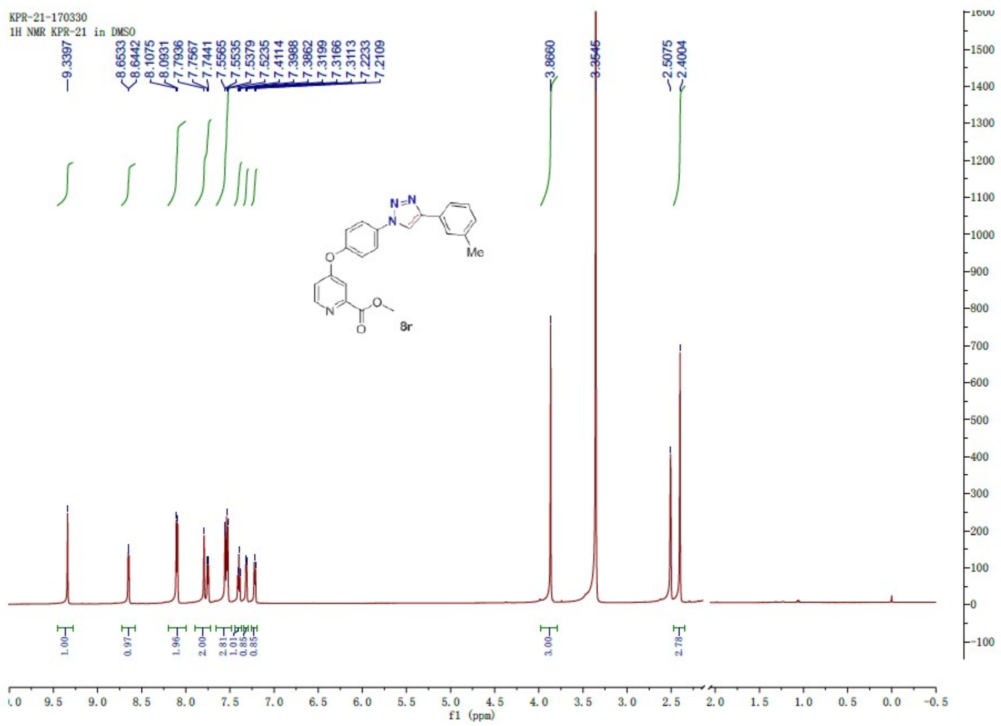
KPR-21-170330
1H NMR KPR-21 in DMSO
8r
f1 (ppm)

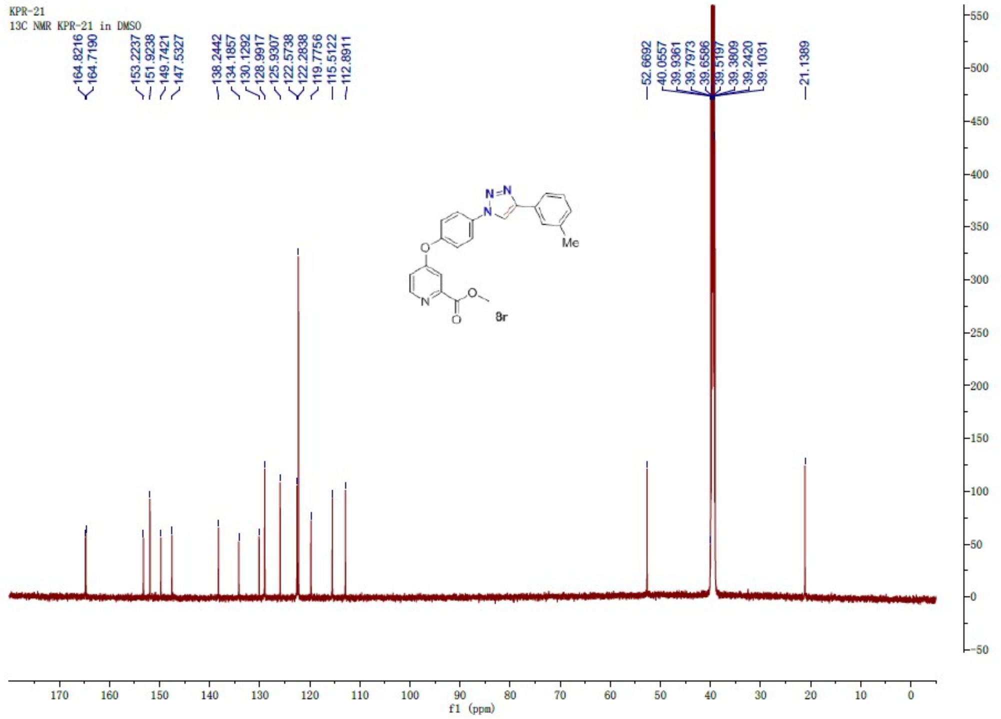
KPR-21
13C NMR KPR-21 in DMSO
8r
f1 (ppm)

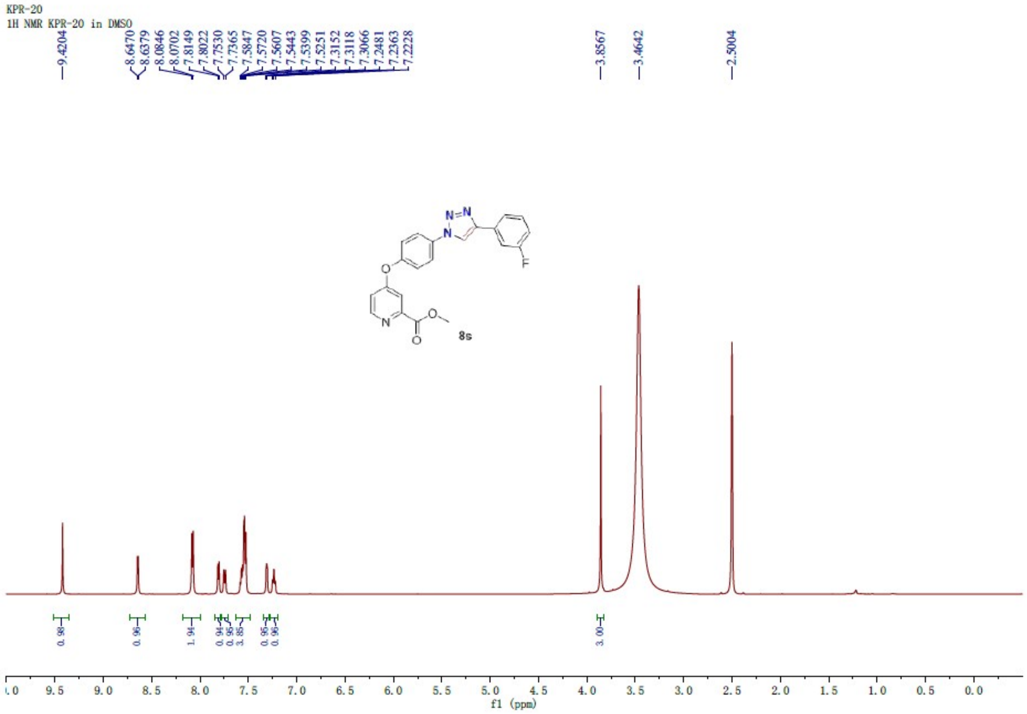
KPR-20
1H NMR KPR-20 in DMSO
9.4204
8.6470
8.6379
8.0846
8.0702
7.8149
7.8022
7.7530
7.7365
7.5847
7.5720
7.5607
7.5443
7.5399
7.5251
7.3152
7.3118
7.3066
7.2481
7.2363
7.2228
3.8567
3.4642
2.5004
N=N
N
O
F
N
O
O
8s
0.98
0.96
1.94
0.94
0.95
3.85
0.95
0.96
3.00
9.5
9.0
8.5
8.0
7.5
7.0
6.5
6.0
5.5
5.0
4.5
4.0
3.5
3.0
2.5
2.0
1.5
1.0
0.5
0.0
f1 (ppm)

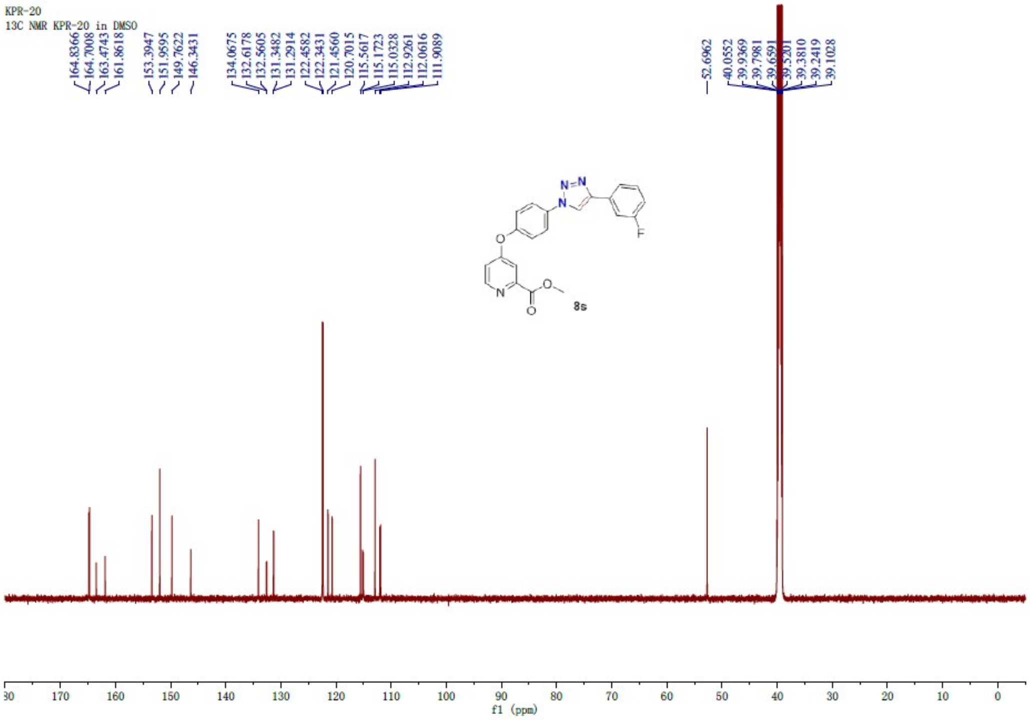
KPR-20
13C NMR KPR-20 in DMSO
164.8366
164.7008
163.4743
161.8618
153.3947
151.9595
149.7622
146.3431
134.0675
132.6178
132.5605
131.3482
131.2914
122.4582
122.3431
121.4560
120.7015
115.5617
115.1723
115.0328
112.9261
112.0616
111.9089
52.6962
40.0552
39.9369
39.7981
39.6591
39.5201
39.3810
39.2419
39.1028
N=N
N
O
F
N
O
O
8s
170
160
150
140
130
120
110
100
90
80
70
60
50
40
30
20
10
0
f1 (ppm)

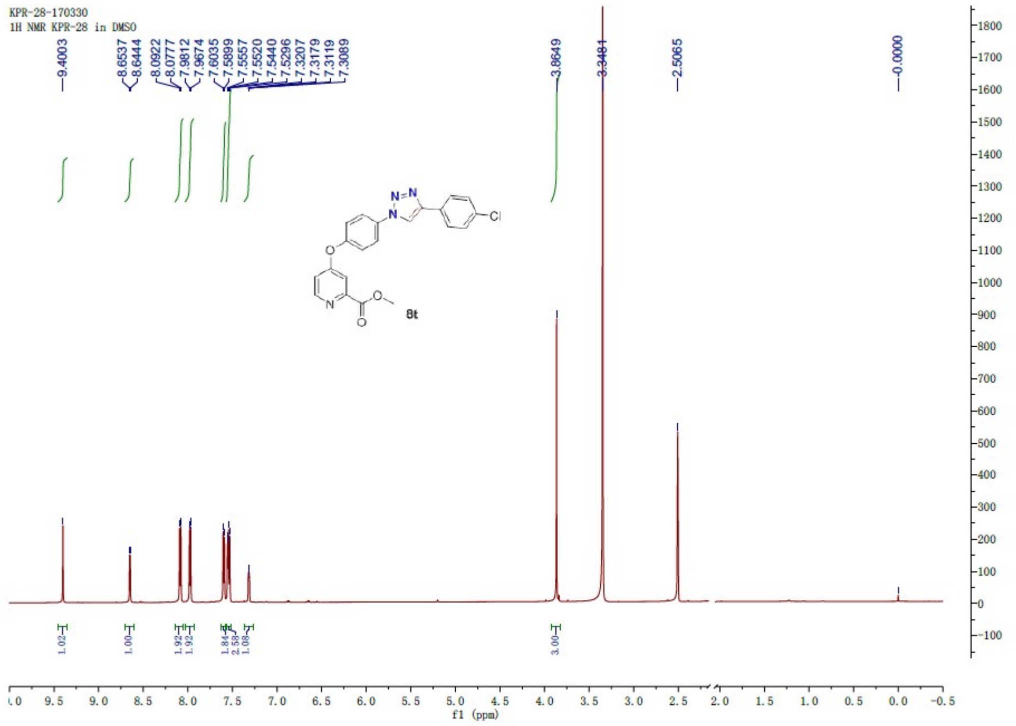
KPR-28-170330
1H NMR KPR-28 in DMSO
9.4003
8.6537
8.6444
8.0922
8.0777
7.9812
7.9674
7.6035
7.5899
7.5557
7.5520
7.5440
7.5296
7.3207
7.3179
7.3119
7.3089
3.8649
3.3481
2.5065
-0.0000
8t
1.02
1.00
1.92
1.92
1.84
2.58
1.08
3.00
f1 (ppm)

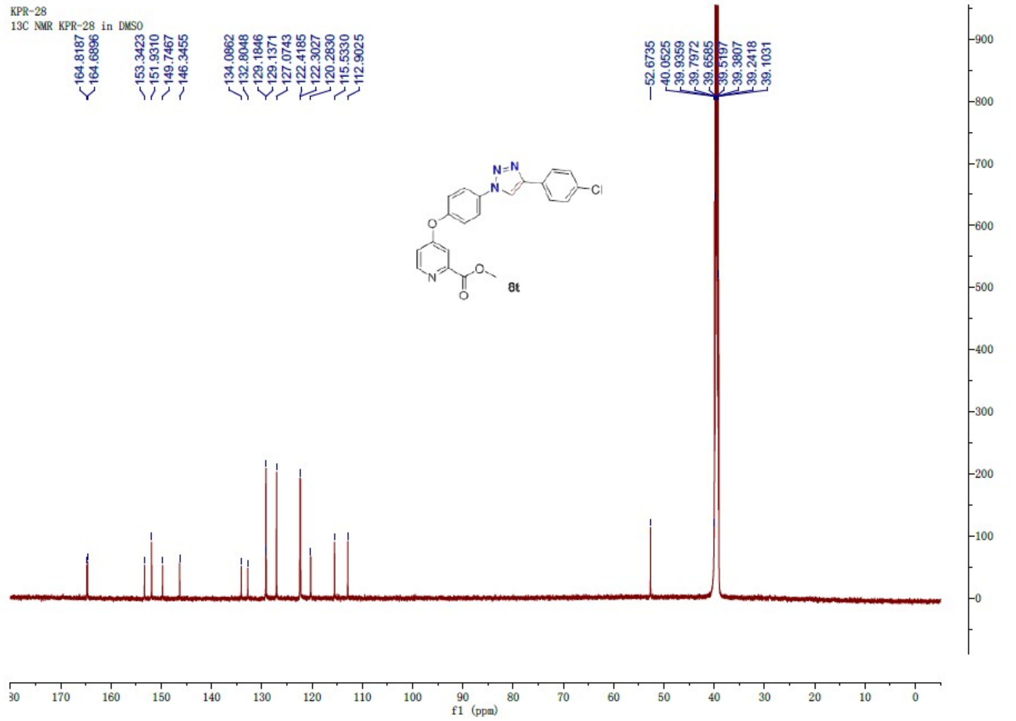
KPR-28
13C NMR KPR-28 in DMSO
164.8187
164.6896
153.3423
151.9310
149.7467
146.3455
134.0862
132.8048
129.1846
129.1371
127.0743
122.4185
122.3027
120.2830
115.5330
112.9025
52.6735
40.0525
39.9359
39.7972
39.6585
39.5197
39.3807
39.2418
39.1031
8t
f1 (ppm)

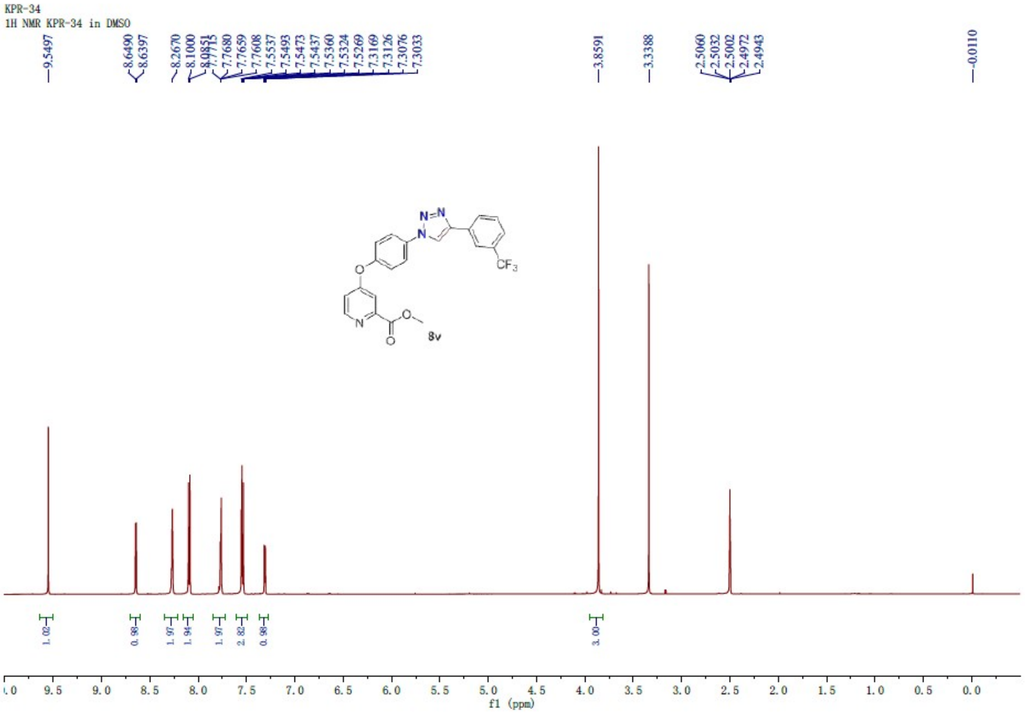
KPR-34
1H NMR KPR-34 in DMSO
9.5497
8.6490
8.6397
8.2670
8.1000
7.7680
7.7659
7.7608
7.5537
7.5493
7.5473
7.5437
7.5360
7.5324
7.5269
7.3169
7.3126
7.3076
7.3033
3.8591
3.3388
2.5060
2.5032
2.5002
2.4972
2.4943
-0.0110
CF3
8v
1.02
0.98
1.97
1.94
1.97
2.82
0.98
3.00
f1 (ppm)

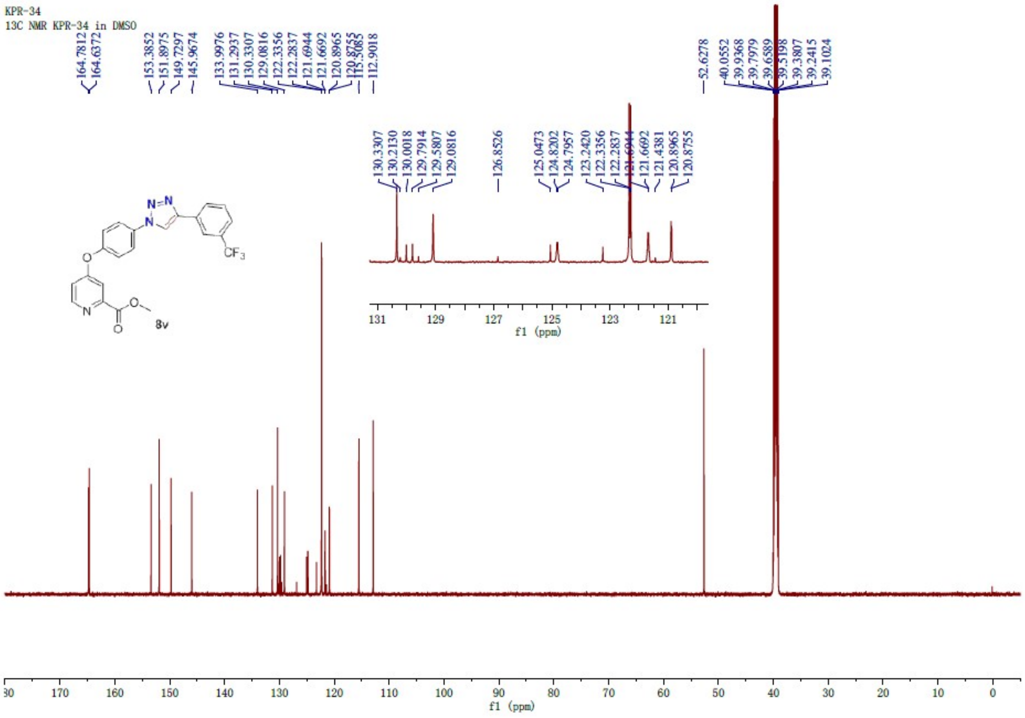
KPR-34
13C NMR KPR-34 in DMSO
164.7812
164.6372
153.3852
151.8975
149.7297
145.9674
133.9976
131.2937
130.3307
129.0816
122.3356
122.2837
121.6944
121.6692
120.8965
112.9018
52.6278
40.0552
39.9368
39.7979
39.6589
39.5198
39.3807
39.2415
39.1024
130.3307
130.2130
130.0018
129.7914
129.5807
129.0816
126.8526
125.0473
124.8202
124.7957
123.2420
122.3356
122.2837
121.6692
121.4381
120.8965
120.8755
CF3
8v
f1 (ppm)

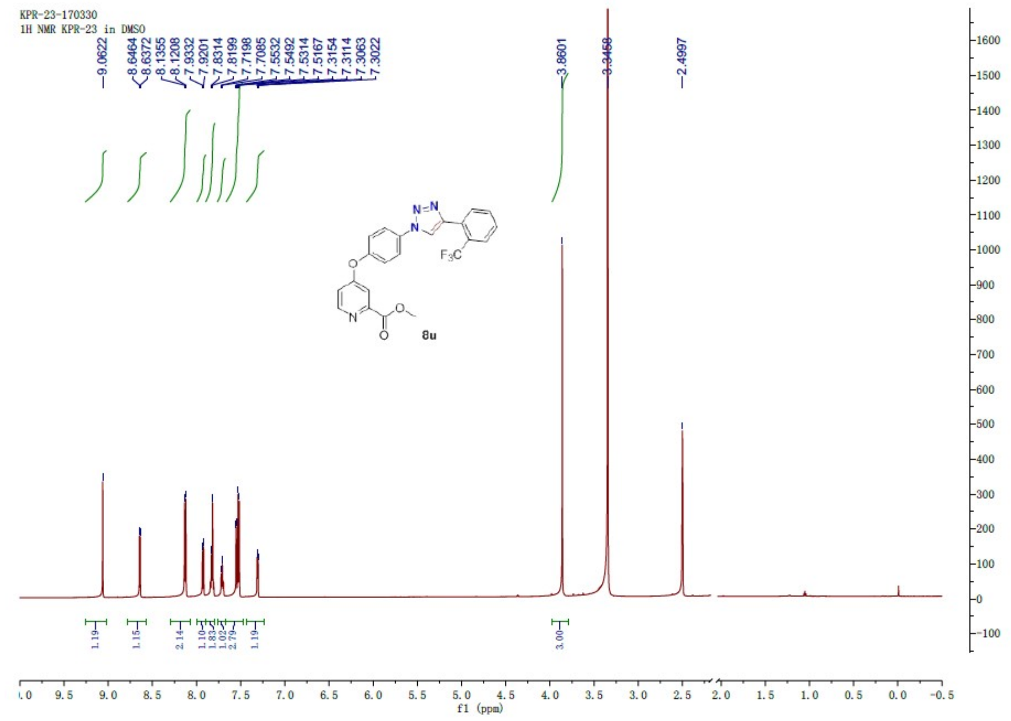
KPR-23-170330
1H NMR KPR-23 in DMSO
9.0622
8.6464
8.6372
8.1355
8.1208
7.9332
7.9201
7.8314
7.8199
7.7198
7.7085
7.5532
7.5492
7.5314
7.5167
7.3154
7.3114
7.3063
7.3022
3.8601
3.3458
2.4997
8u
f1 (ppm)

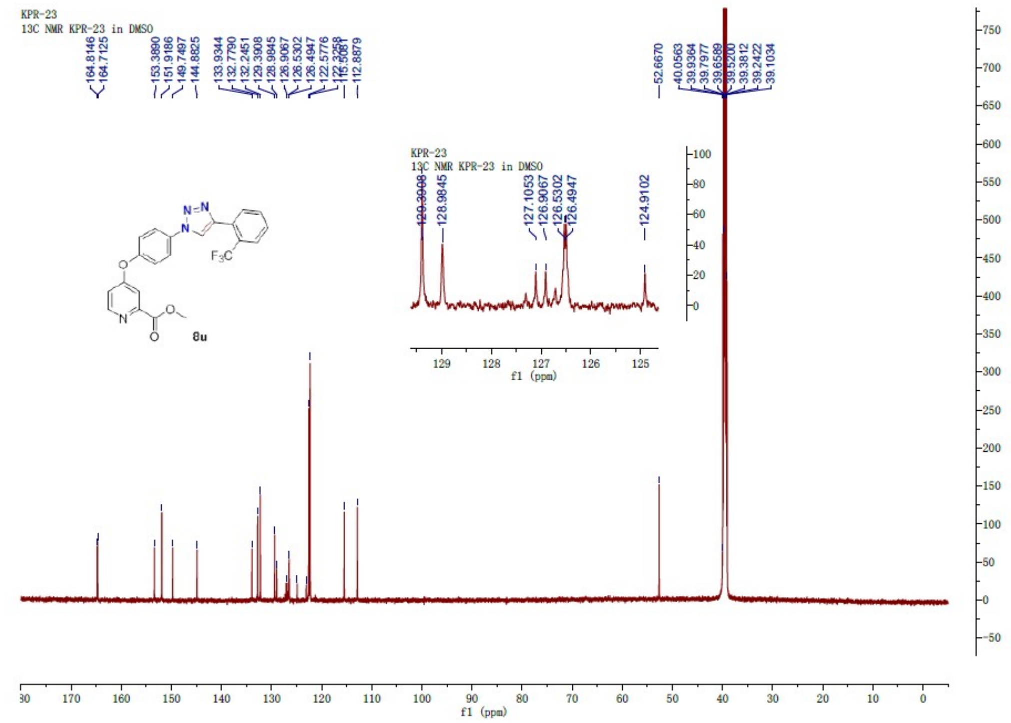
KPR-23
13C NMR KPR-23 in DMSO
164.8146
164.7125
153.3890
151.9186
149.7497
144.8825
133.9344
132.7790
132.2451
129.3908
128.9845
126.9067
126.5302
126.4947
122.5776
112.8879
52.6670
40.0563
39.9364
39.7977
39.6589
39.5200
39.3812
39.2422
39.1034
KPR-23
13C NMR KPR-23 in DMSO
129.3908
128.9845
127.1053
126.9067
126.5302
126.4947
124.9102
8u
f1 (ppm)

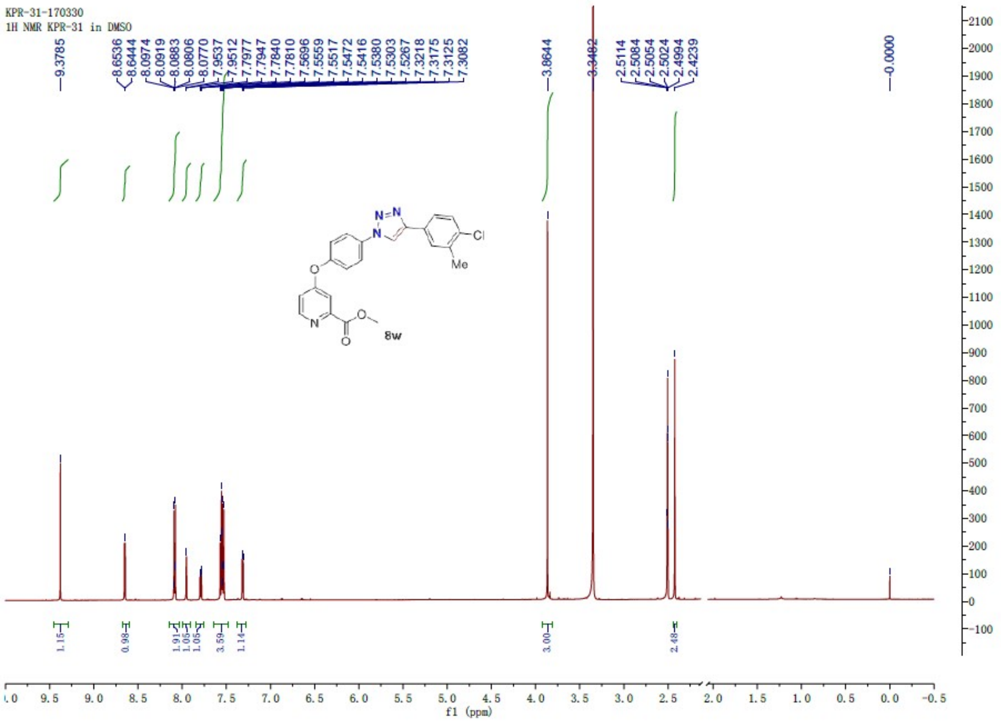
KPR-31-170330
1H NMR KPR-31 in DMSO
8w
f1 (ppm)

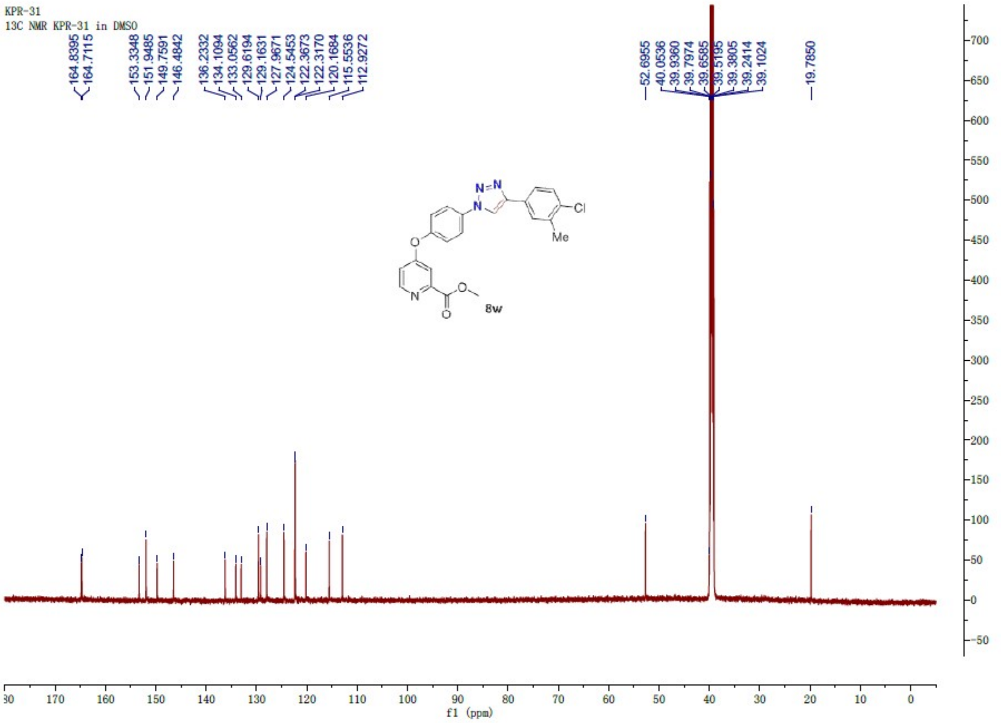
KPR-31
13C NMR KPR-31 in DMSO
8w
f1 (ppm)

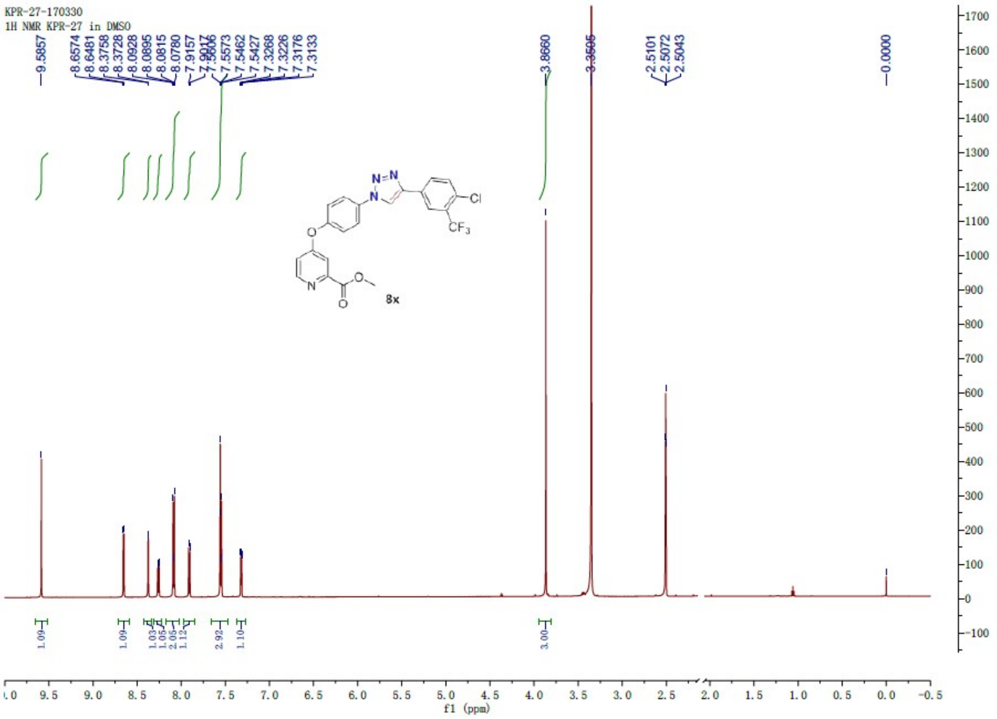
KPR-27-170330
1H NMR KPR-27 in DMSO
8x
f1 (ppm)

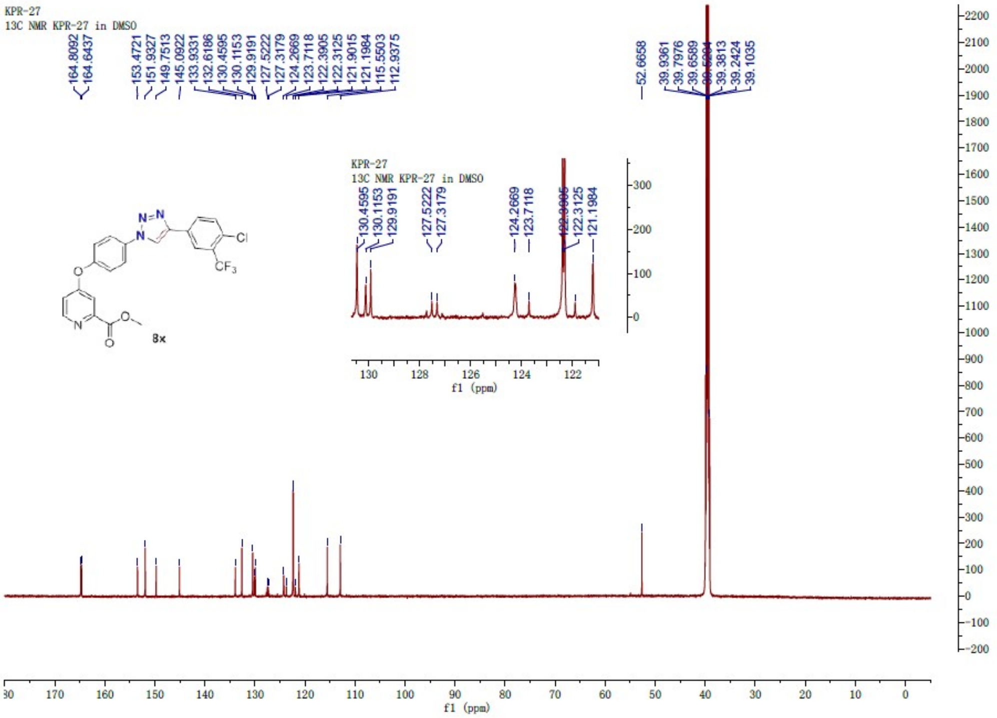
KPR-27
13C NMR KPR-27 in DMSO
8x
f1 (ppm)

KPR-22-170330
1H NMR KPR-22 in DMSO

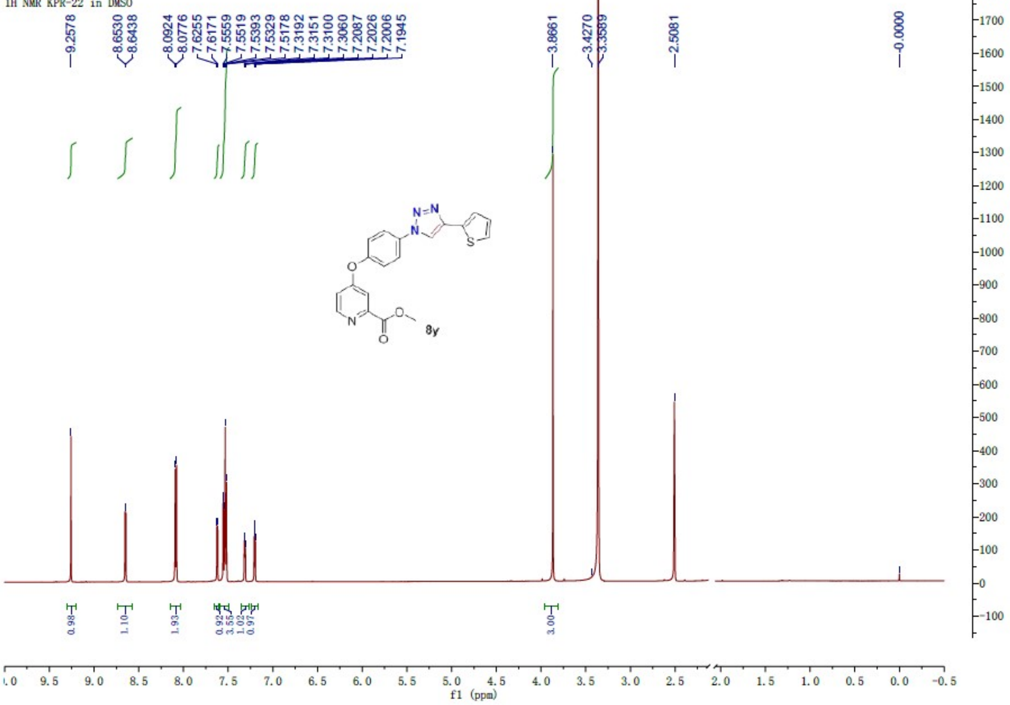

KPR-22
13C NMR KPR-22 in DMSO

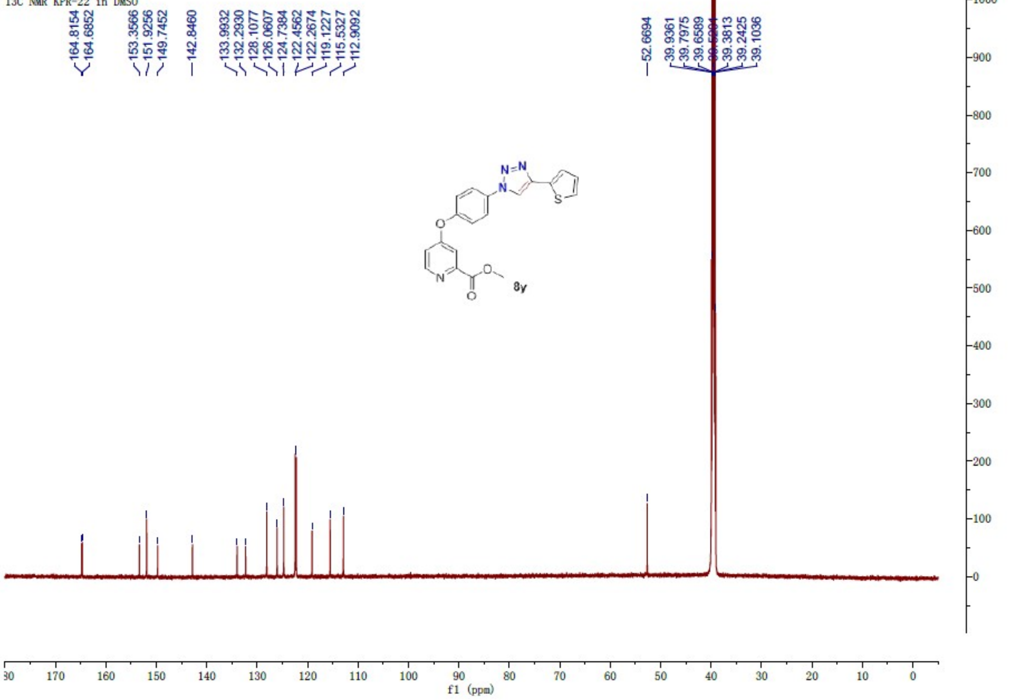

Supplement: Supplementary file 1 [file molecules-22-01759-s001.pdf]
